# Pre-Emptive Priming of Human Skin Improves Cutaneous Scarring and Is Superior to Immediate and Delayed Topical Anti-Scarring Treatment Post-Wounding: A Double-Blind Randomised Placebo-Controlled Clinical Trial

**DOI:** 10.3390/pharmaceutics13040510

**Published:** 2021-04-08

**Authors:** Sara Ud-Din, Traci A. Wilgus, Douglas D. McGeorge, Ardeshir Bayat

**Affiliations:** 1 Plastic and Reconstructive Surgery Research, NIHR Manchester Biomedical Research Centre, University of Manchester, Manchester M13 9PT, UK; Sara.ud-din@manchester.ac.uk; 2 Department of Pathology, Wexner Medical Center, Ohio State University, Columbus, OH 43210, USA; traci.wilgus@osumc.edu; 3 Grosvenor Nuffield Hospital, Chester CH4 7QP, UK; douglas@douglasmcgeorge.com; 4 MRC-SA Wound Healing Unit, Division of Dermatology, University of Cape Town, Cape Town 7925, South Africa

**Keywords:** pre-emptive skin priming, zonal therapy, human skin, cutaneous scarring, topical treatment, surgical wounds, wound healing, mast cells, angiogenesis, EGCG, green tea active, polyphenol

## Abstract

The concept of pre-emptive priming of skin pre-surgery offers a novel approach in optimizing cutaneous scarring outcome. We previously showed an anti-scarring topical (epigallocatechin-3-gallate (EGCG)) is effective in improving skin scarring when applied post-surgery. The objective was to deliver an active compound at the optimal time in order to maximize its impact and improve cutaneous scarring. Therefore, pre-emptive application of anti-scarring topical pre-surgery compared with post-surgery can potentially be superior on scarring outcome. This double-blinded randomized placebo-controlled trial compares the effects of pre-emptive priming of skin with an anti-scarring topical pre-surgery versus post-surgery. Healthy volunteers (*n* = 40) were split into 4-groups; each undergoing different modes of application versus placebo: Group-1 = priming (7Days) pre-injury, Group-2 = priming (3D) pre-injury, Group-3 = immediate (0D) day-of-injury, Group-4 = delayed application (14D) post-injury. Excisional skin-biopsies in upper-arms were evaluated weekly with multiple quantitative devices over 8-weeks. Histological, immunohistochemical, mRNA sequencing and QRT-PCR studies were performed on tissue-biopsies. EGCG reduced mast cells at weeks-4 and 8 by gene and protein analyses (*p* < 0.01). Group 1 was superior to other groups (*p* < 0.01) in both clinical (blood flow) and laboratory parameters (elastin and immune marker expression). Additionally, there was down-regulation of angiogenic-markers by mRNA-sequencing and of CD31 and VEGF-A at weeks-4 and 8 (*p* < 0.01) by immunohistochemistry and at week-4 (*p* < 0.05) by QRT-PCR. EGCG increased antioxidant levels (HO-1) at week-4 (*p* < 0.01) plus elastin at week-8 (*p* < 0.01). In conclusion, pre-emptive priming of skin pre-injury has significant beneficial effects on surgically induced skin scarring shown by reducing mast cells, blood flow and angiogenesis plus increasing elastin content. This clinical trial was registered with ISRCTN (ISRCTN70155584).

## 1. Introduction

Wound healing is a complex process that aims to restore the integrity of the skin as quickly as possible [1,2]. This is divided into four overlapping phases including hemostasis, inflammation, proliferation and remodeling of collagen by fibroblasts with many interactions between fibrotic and anti-fibrotic growth factors ultimately leading to scar formation [1]. Skin scars are the inevitable outcome of dermal tissue repair following full thickness cutaneous injury. An estimated 100 million patients acquire permanent skin scars in the developed world post-elective surgery each year alone [1]. Some of these skin scars may heal poorly and become clinically and pathologically abnormal by becoming symptomatic and may even develop into hypertrophic or keloid scars [2]. Therefore, optimal evidence-based scar management is essential. Nevertheless, most current treatment strategies of the initial steps for the management of a newly formed skin scar often adopt a watch-and-wait approach prior to commencing targeted therapy [3]. The ideal approach, however, we argue here, is to do the opposite and take an early intervention prior to surgically induced skin injury in order to minimize the risk of developing a poor scar outcome. This is particularly relevant for those individuals undergoing elective/scheduled surgery as it is routinely arranged in advance, otherwise known as skin trauma by prior appointment as opposed to skin trauma by accident.

There have been a number of strategies used to minimize skin scarring including the use of tissue engineering [4,5] and biomaterial-based dressings. Transdermal patches have had beneficial effects in accelerating wound repair and inhibiting scar formation [6]. There have been a lack of studies using these modalities for the prevention of scarring prior to injury, however, in vitro studies have demonstrated transdermal patches have the potential to inhibit hypertrophic scar formation [7,8,9]. Some recent studies support the concept of priming the skin prior to an invasive intervention for achieving an optimal result [10,11,12,13,14,15]. However, the concept of pre-emptively priming the skin prior to surgically induced injury has not been thoroughly evaluated. Specifically, a gap in knowledge remains regarding the use of topical priming agents and their effect on skin scarring.

We previously demonstrated using a double-blind randomized controlled clinical-trial the concept of immediate versus delayed application of a topical formulation post-surgical wounding in an excisional punch-biopsy model [16]. The objective was to deliver an active compound at the optimal time post-surgically induced injury, in order to maximize its impact and improve healing. Indeed, we demonstrated reduced scar thickness and angiogenesis plus increased hydration and elasticity when an anti-scarring topical formulation (epigallocatechin-3-gallate (EGCG)) was applied immediately to the zone of injury compared with delayed application of topical two-weeks post-wounding. EGCG has attracted interest due to its range of biological effects as it is known to have potent anticarcinogenic, antimicrobial, anti-aging, anti-angiogenic and anti-inflammatory properties [16,17,18] as well as increasing antioxidant activity and enhancing gap junctional communication between cells leading to a protective role for tumor development [19]. Evidence has suggested EGCG inhibits tumor progression and improves inflammatory diseases [20,21] whilst also having antiviral and antibacterial effects [22,23]. EGCG has also been shown to potentially play a role in preventing fibrosis by inhibiting expression of vascular endothelial growth factor (VEGF), transcription growth factor beta 1 (TGF-β1) and connective tissue growth factor (CTGF) in a number of organs [24,25]. Furthermore, it has inhibited growth and induced shrinkage in keloid scar tissues [26] and reduced inflammatory and fibrotic markers in normal scars ex vivo [27]. These observations led us to hypothesize that EGCG may be a potential candidate for use as a topical agent in the treatment of skin scarring in both studies.

**Figure 1:**
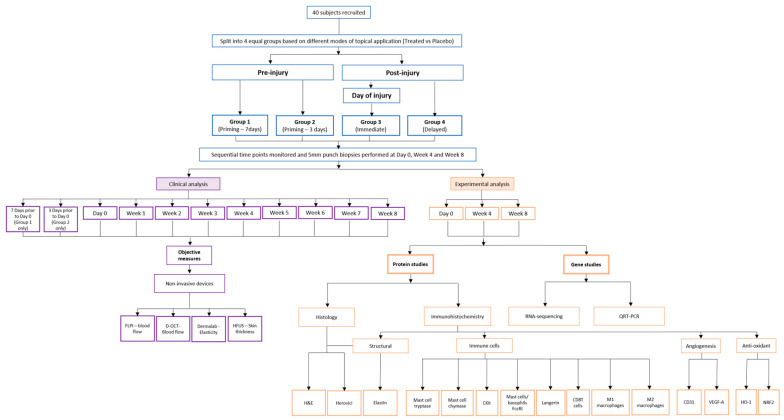
Study Methodology. A flowchart to demonstrate the methodology of the study including both the pre-injury priming versus immediate and post-injury timing of the topical application modalities, study time points and the non-invasive and invasive measures used.

The findings from the previous study provoked the hypothesis that earlier application of an anti-scarring topical to the planned anatomical site of surgery could have beneficial effects on the outcome of scarring. Therefore, we postulated that pre-emptive priming with anti-scarring topical to the zone of injury, prior to performing surgically induced excisional punch-biopsies, could further maximize the effects by targeting the source of inflammation earlier. Therefore, to further explore this concept, a double-blind randomized placebo-controlled trial was conducted using a temporal punch-biopsy model (Figure 1). Various modes of anti-scarring topical formulation (EGCG) application were evaluated in this well-established human skin scarring model utilizing a full-thickness excisional surgical biopsy approach to identify whether pre-emptive priming pre-surgically induced injury had a greater impact on scarring outcome compared to day of- or post-injury application.

## 2. Materials and Methods

### 2.1. Demographics

**Figure 2:**
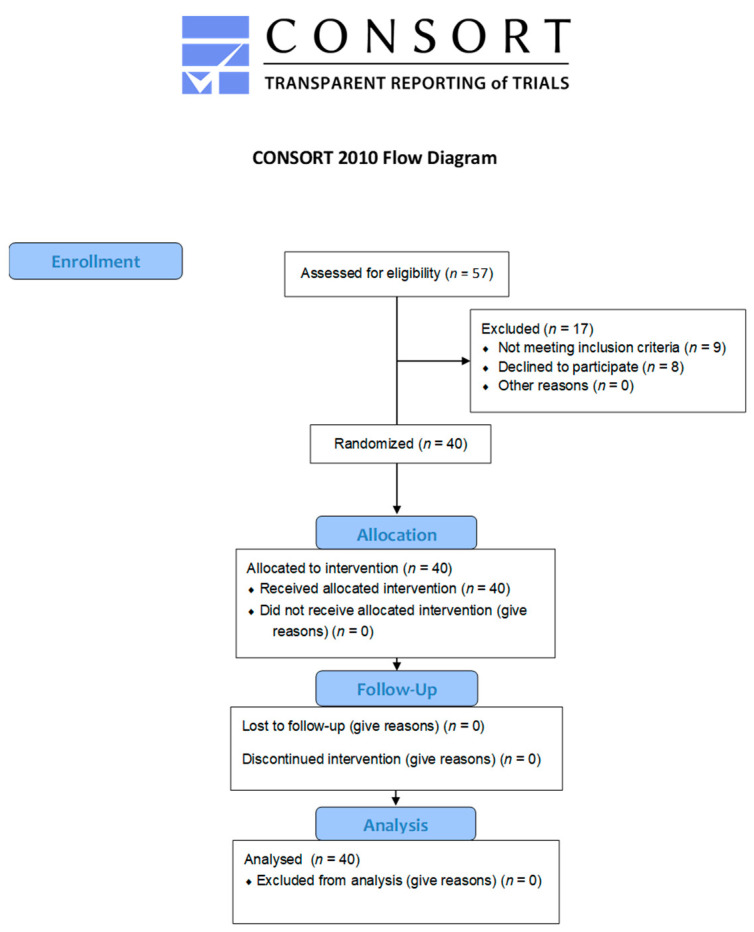
Consort Flow Diagram.

All demographic information is outlined in Table 1. Participant Consort flow can be found in Figure 2 and Consort Checklist in Supplementary Material Table S1. 

### 2.2. Protocol

Inclusion and exclusion criteria are the same as in Ud-Din et al. [16]. This study was performed in line with the Declaration of Helsinki principles. University of Manchester Research Ethics Committee and Trust Research and Development Department granted approvals (UREC Reference: 14333). Forty healthy volunteers were recruited, provided written informed consent and were followed up at Manchester University NHS Foundation Trust, UK from 17 June 2019 until 12 September 2019). The trial was registered as International Standard Randomized Controlled Trial (ISRCTN 70155584) on 4 June 2020.

### 2.3. Assignment and Masking

An independent statistician from the Manchester University NHS Foundation Trust determined the sample size of 40 participants to have 80% power to detect a large effect size of 2 (mean difference/common standard deviation) for the primary outcome using an independent samples *t*-test with a 5% two-sided significance level. Randomization was conducted by arm in nQuery Advisor 7.0 with a computer-generated permuted block design with mixed block sizes and random seed. A member of the team who was not involved in this study held the randomization list and labelled the topical tubes to ensure the researcher was blinded throughout the study. Researcher and participants were blinded as to which topicals (EGCG or placebo) were assigned to which arm throughout all follow-up appointments until completion of study.

### 2.4. Participant Flow and Follow-Up

**Figure 3:**
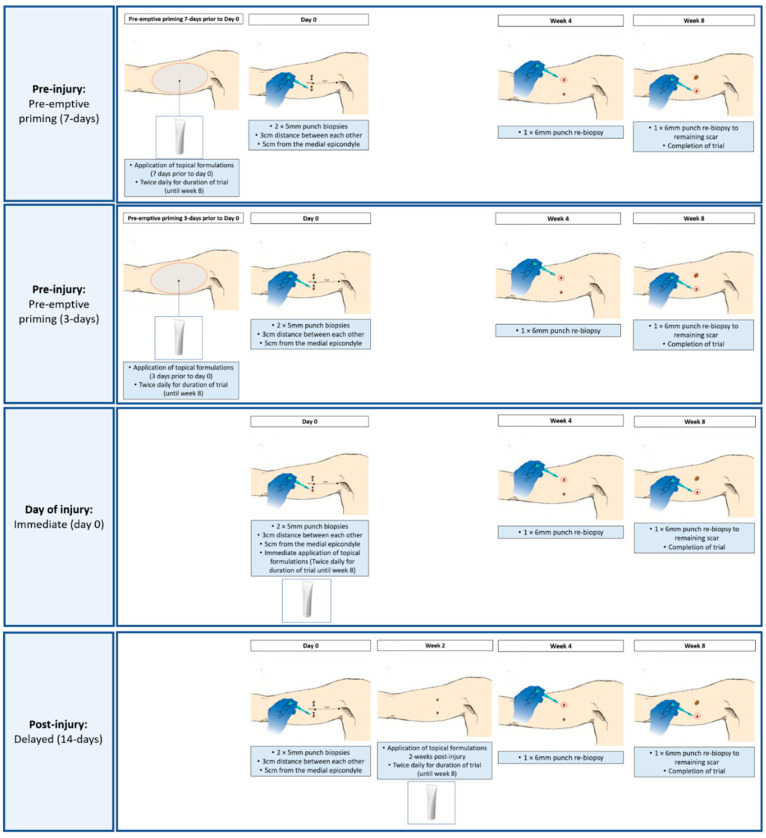
Punch biopsy methodology. Diagrammatic representation of the methodology for each of the groups (Pre-injury: pre-emptive priming (7-days), pre-injury: pre-emptive priming (3-days), day of injury (immediate application on day 0), post-injury (delayed 2 weeks post injury) including biopsy time points and time of topical applications. Each group applied both topical formulations twice daily for the duration of the study until week 8, with only the starting time point differing between groups.

To further understand the role of EGCG in pre-emptive priming of the wound site, a double-blind randomized placebo-controlled trial was conducted using temporal punch biopsy model over longer time points of 8 weeks (Figure 3).

Clinical non-invasive quantitative devices were used to objectively monitor healing at weekly intervals until week 8 and punch biopsies were performed on day 0 (uninjured skin) and at weeks 4 and week 8 for protein and gene expression analyses. All participants had two wounds created in both arms with one arm receiving EGCG and the other arm placebo formulation. Randomization was based on which arm received which treatment which also accounted for right- or left-hand dominance. Topical EGCG and a placebo topical were applied to the scars twice daily until week 8. The week 4 time point was chosen based on our previous main finding of reduced mast cell count and week 8 time point was chosen to identify any later effects with any structural changes in the scars. Participants were split into 4 groups (*n* = 10 in each group) in order to compare different modes of topical application:

- Group 1: Pre-emptive Priming (7-days)—Application of topicals 7 days prior to initial biopsies on day 0
- Group 2: Pre-emptive Priming (3-days)—Application of topicals 3 days prior to initial biopsies on day 0
- Group 3: Immediate delivery (0-day)—Immediate application of topicals on day 0 (initial biopsies)
- Group 4: Delayed delivery (14-days)—Delayed application of topicals 2 weeks post-initial biopsies

All groups continued twice daily topical applications until week 8 completion of trial, with only the starting time point differing between groups as outlined above. (Groups 1 and 2 only) 7 days or 3 days prior to Day 0: Groups 1 and 2 were given EGCG topical and a placebo topical (same base ingredients but without the active EGCG) to commence application twice daily to the zone of injury where the biopsies would be taking place on Day 0 for the duration of study (until week 8). Topicals were packaged identically, with labels indicating left or right arm. On Day 0, all 40 participants (all groups) had two 5-mm-diameter full-thickness skin biopsies performed under local anesthetic using 1% lidocaine to both upper inner arms. This was positioned 5 cm from the axillary hairline and parallel to the medial epicondyle and were 3 cm apart from each other on each arm. The biopsy wound sites were dressed with Kaltostat (ConvaTec, Deeside, Chester, UK), gauze, and Tegaderm plus pad dressing (3 M, Minneapolis, MN). Participants were asked to ensure that the dressings remained in situ for 48 h only, and then no further dressings were required, and wounds were left exposed to the air. Group 3 commenced topical applications on Day 0 for the duration of study (until week 8) by applying the topical around the dressing site for the first 48 h and then continuing. Group 4 commenced topicals on day 14 (2 weeks after the initial biopsies when a scar had formed) for the duration of study (until week 8). Participants had weekly follow-up appointments where non-invasive measurements were taken. On week 4 and week 8, 6-mm diameter re-biopsies were performed. The biopsies were chosen for each time point prior to commencement of the stud and all participants had the same biopsy location for week 4 and week 8 time points. The biopsy wound sites were dressed as above. Additionally, all wounds were monitored at each visit and healed successfully in each group. Topical application compliance was assessed on a weekly basis and participants were asked to complete a daily diary.

### 2.5. Objective Non-Invasive Quantitative Devices

Objective non-invasive devices were performed at every time point for all participants in each group (Table 2).

### 2.6. Laboratory Techniques

#### 2.6.1. Immunohistochemical Analysis

Biopsy samples of scar and uninjured tissues were formalin fixed and paraffin embedded and then sectioned at a thickness of 5 μm. Sectioned were mounted on glass slides with samples in duplicate. Slides were dewaxed in xylene and graded ethanol and antigen retrieval using citrate buffer pH 6 was performed. Immunohistological slides were carried out using the Leica Novolink (Milton Keynes, UK) peroxidase staining kit protocol and were incubated overnight at 4 degrees centigrade. Immunofluorescence stains were performed having been washed with phosphate buffered saline/0/01% Teen 20 and subsequent blocking with 10% normal goat serum in PBST followed by the primary antibodies overnight at 4 degrees centigrade. Slides were then counterstained with DAPI. M2 macrophage analysis was conducted using cryosections with acetone fixation, addition of primary antibodies and incubation at 4 degrees centigrade overnight. Same method used as Ud-Din et al., 2019 [16] (Table 3).

#### 2.6.2. RNA Extraction

RNA extraction was performed using the RNeasy mini kit protocol (Qiagen, Hilden, Germany). RNA purity and quantity were assessed by a Nanodrop ND-100, Version 3.0.1 (NanoDrop Technologies, Wilmington, DE, USA). Same method used as Ud-Din et al., 2019 [16].

#### 2.6.3. mRNA Sequencing

Illumina Nova-seq PE150 was used for mRNA sequencing (Novogene Europe, Cambridge, UK). Data has been deposited to GEO (Accession number: GSE152781). For quality control, raw data/reads of FASTQ format were initially processed through in-house scripts. In this step, clean data (clean reads) were obtained by removing reads containing adapter and poly-N sequences and reads with low quality from raw data. All downstream analyses were based on the clean data with high quality. The reference genome and gene model annotation files were taken from NCBI/UCSC/Ensembl (Ensembl version for read alignment: Homo Sapiens GRCh37/hg19). Paired-end clean reads were aligned to the reference genome using the Spliced Transcripts Alignment to a Reference (STAR) software, that uses sequential maximum mappable seed search in uncompressed suffix arrays followed by seed clustering and stitching procedure. HTSeq was used for gene expression quantification to count the read numbers and reads per kilobase of exon model per million mapped reads (RPKM) of each gene was calculated. Differential expression anaysis was performed. (For DESeq2 with biological replicates) Differential expression analysis between two groups (three biological replicates per condition) was performed using DESeq2 R package. *p* values were adjusted using the Benjamini and Hochberg’s approach to account for the False Discovery Rate. Genes with an adjusted *p* value < 0.05 were assigned as differentially expressed. Significant differential expression was determined by a corrected *p*-value of 0.005 and log_2_ (fold change) of 1. Enrichment analysis was performed. Gene Ontology (GO) annotates genes to biological processes, molecular functions, and cellular components in a directed acyclic graph structure, Kyoto Encyclopedia of Genes and Genomes (KEGG) annotates genes to pathways, and Reactome annotates genes to pathways and reactions in human biology. GO enrichment analysis of differentially expressed genes was conducted by GOseq R software. GO terms with corrected *p* value less than 0.05 were considered significantly enriched by differential expressed genes. A list of mRNA sequencing software can be found in Table 4.

#### 2.6.4. QRT-PCR

QRT-PCR was conducted using expression of chosen candidate genes and two reference genes for normalization (Ribosomal protein L32 and succinate dehydrogenase complex, subunit A. 1 μg RNA was used as a template for CDNA synthesis using EvoScript CDNA Synthesis Kit (Roche Biosciences, Basel, Switzerland). PCR was carried out in triplicates using the Roche LightCycler 480 (Roche Diagnostics) and negative control reactions were included. Same method as Ud-Din et al. [16] (Table 5).

### 2.7. Statistics and Data Analysis 

An independent statistician from the Manchester University NHS Foundation Trust determined the sample size of 40 participants to have 80% power to detect a large effect size of 2 (mean difference/common standard deviation) for the primary outcome using an independent samples *t*-test with a 5% two-sided significance level. Clinical data was analysed by independent statisticians using paired *t*-test which was used to compare the mean difference in percentage change from baseline between the arms. The comparison between the 4 groups at each time point was performed using a one-way ANOVA. If the variances were not homogeneous, a Welch’s ANOVA was performed and if the data was non-normally distributed, a Kruskal–Wallis test was performed. In the case where there was a statistically significant difference between the groups, a pairwise comparison was performed to determine the particular group differences. When the pairwise comparison followed a one-way ANOVA, Tukey’s honestly significance difference test post hoc test was used and when it followed a Welch’s ANOVA, a Games-Howell post hoc test was used. Definiens Tissue Studio software version 64.4.0 (Definiens, Munich, Germany) was performed to analyze quantitative stains for both scar tissue and uninjured tissues. For each separate stain, the whole tissue sections were scanned in duplicate, ensuring that the exposure settings were consistent for all slides to account for variability between samples. Analysis of Definiens data followed the above method. Analysis of QRT-PCR data was performed using paired *t*-test with significant differences defined as *p* < 0.05. QRT-PCR data was expressed as fold change (2−(ΔΔCT). For clinical and immunohistochemical analyses, significances were defined as *p* ≤ 0.01 to account for multiple testing with Bonferroni correction and significances as *p* < 0.05 have also been included for interest. 

## 3. Results

### 3.1. Mast Cell Reduction Is Most Significant in Pre-Injury Priming Compared to Immediate and Delayed Topical Anti-Scarring Application 

**Figure 4:**
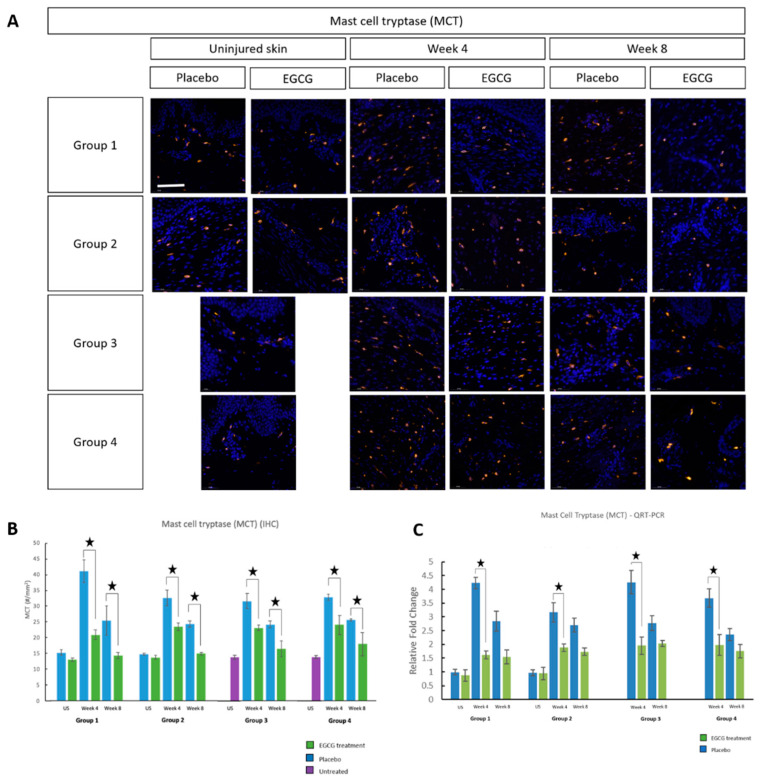
Mast cell tryptase (MCT) analysis of topical epigallocatechin-3-gallate (EGCG) versus placebo. (**A**) MCT immunohistochemical marker images demonstrate a reduction with EGCG in all groups compared to placebo at weeks 4 and 8. (**B**) MCT measurements (#/mm^2^) showed that EGCG-treated scars were significantly lower at weeks 4 and 8 compared to placebo (*p* < 0.01) for all groups. Between group analysis demonstrated a significant difference at week 4 (Anova *p* = 0.001) and this was identified as Priming Group 1 (7 days priming) with the greatest differences compared to Group 2 (*p* = 0.002), Group 3 (*p* = 0.003) and Group 4 (*p* = 0.005). (**C**) Quantitative real-time reverse transcriptase–PCR (QRT-PCR) analysis confirmed down-regulation of MCT (*p* < 0.05) in EGCG samples compared to placebo at week 4 in all groups. Significance: ★ *p* ≤ 0.01. Error bars: mean ± SD. Scale bars = 50 μm. (DAPI = blue, MCT = orange).

**Figure 5:**
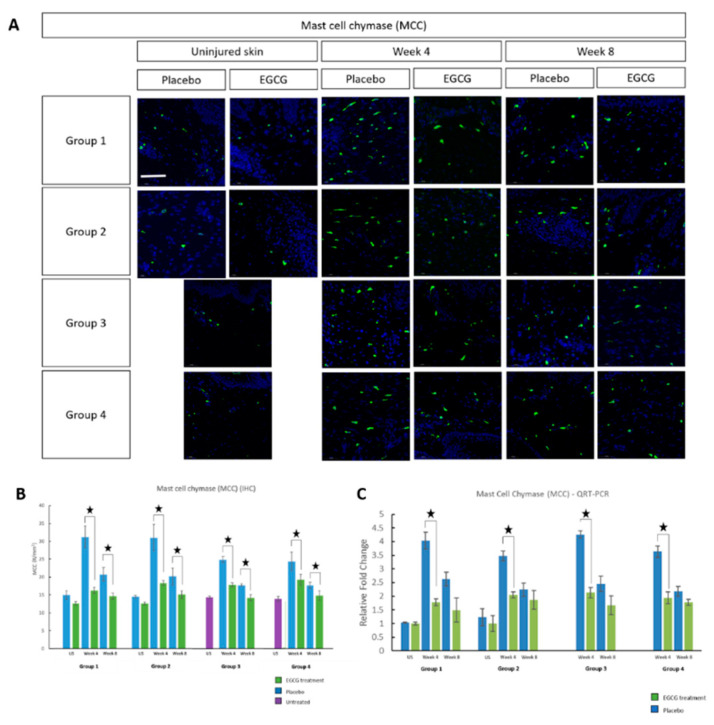
Mast cell chymase (MCC) analysis of topical epigallocatechin-3-gallate (EGCG) versus placebo. (**A**) MCC images show a reduction at weeks 4 and 8 in EGCG samples compared to placebo in all groups. (**B**) MCC measurements (#/mm^2^) showed EGCG samples were significantly lower compared to placebo at weeks 4 and 8 (*p* < 0.01) in all groups. Between groups there were significant differences at week 4 (Anova *p* = 0.001). Group 1 had a greater difference than Group 3 (*p* = 0.01) and Group 4 (*p* = 0.001), and Group 2 had a larger difference than Group 4 (*p* = 0.009). (**C**) Subsequent QRT-PCR analysis confirmed down-regulation of MCC (*p* < 0.05) in EGCG samples compared to placebo at week 4 in all groups. Significance: ★ *p* ≤ 0.01. Error bars: mean ± SD. Scale bars = 50 μm. (DAPI = blue, MCC = green).

**Figure 6:**
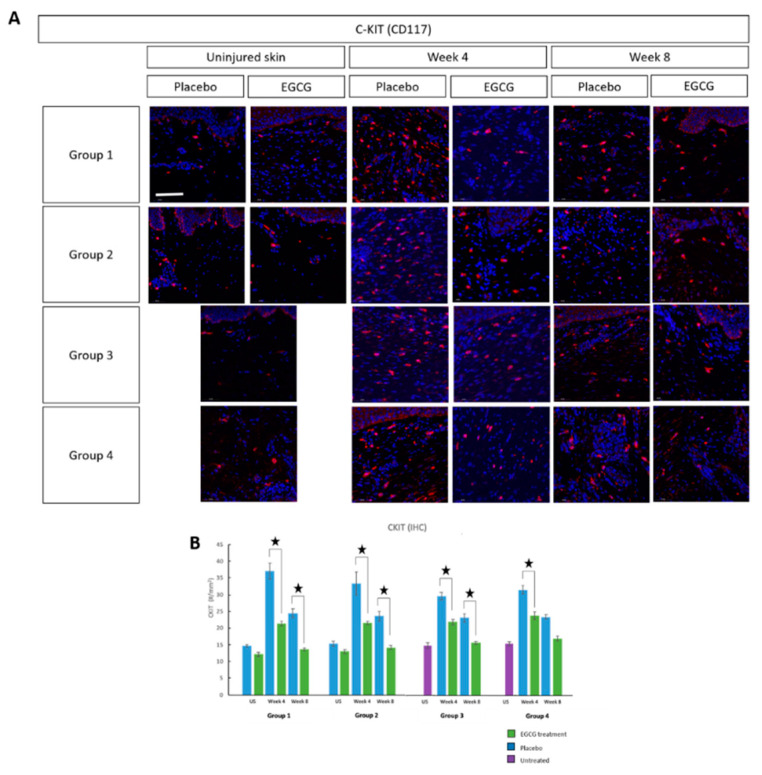
CKit analysis of topical epigallocatechin-3-gallate (EGCG) versus placebo. (**A**) CKit staining images showed a reduction in mast cells with EGCG compared to placebo. (**B**) Further analysis showed a significant difference between EGCG and placebo samples measurements (#/mm^2^) at weeks 4 and 8 (*p* < 0.01) and between the groups at week 4 (Anova *p* = 0.01). The greatest difference was in Group 1 compared to Group 3 (*p* = 0.02) and Group 4 (*p* = 0.02). Significance: ★ *p* ≤ 0.01. Error bars: mean ± SD. Scale bars = 50 μm. (DAPI = blue, CKit = red).

Mast cell identification was performed using mast cell tryptase (MCT) (Figure 4), mast cell chymase (MCC) (Figure 5) and CKit markers (Figure 6). All three markers demonstrated that EGCG-treated scars had significantly fewer positive cells at weeks-4 and 8 compared to placebo-treated scars (*p* < 0.01) for all groups. Placebo values in the pre-emptive groups were higher than in other groups and this may have been due to the act of massage when applying the topicals earlier. Further analysis showed differences between the groups in relation to mast cell reduction. MCT analysis demonstrated a significant difference between groups at week-4 (*p* = 0.001) and the pre-emptive priming group-1 was superior. The percentage differences between the change in placebo and EGCG for group-1 compared to group-2 was 58% (*p* = 0.002), group-3 was 53% (*p* = 0.003) and group-4 was 52% (*p* = 0.005). MCC showed a significant difference at week-4 between groups (*p* = 0.001). Group-1 had a greater difference than group-3 by 29% (*p* = 0.01), group-4 by 41% (*p* = 0.001), and group-2 had a larger difference than group-4 by 29% (*p* = 0.009). CKit analysis further demonstrated a significant difference between the groups at week-4 (*p* = 0.01). The greatest difference was in group-1 compared to group-3 by 26% (*p* = 0.02) and group-4 by 27% (*p* = 0.02). Subsequent QRT-PCR analysis further confirmed down-regulation of MCT and MCC (*p* < 0.05) in EGCG-treated samples at week-4 in all groups.

### 3.2. Langerin Reduction Was Most Significant in Pre-Injury Priming

**Figure 7:**
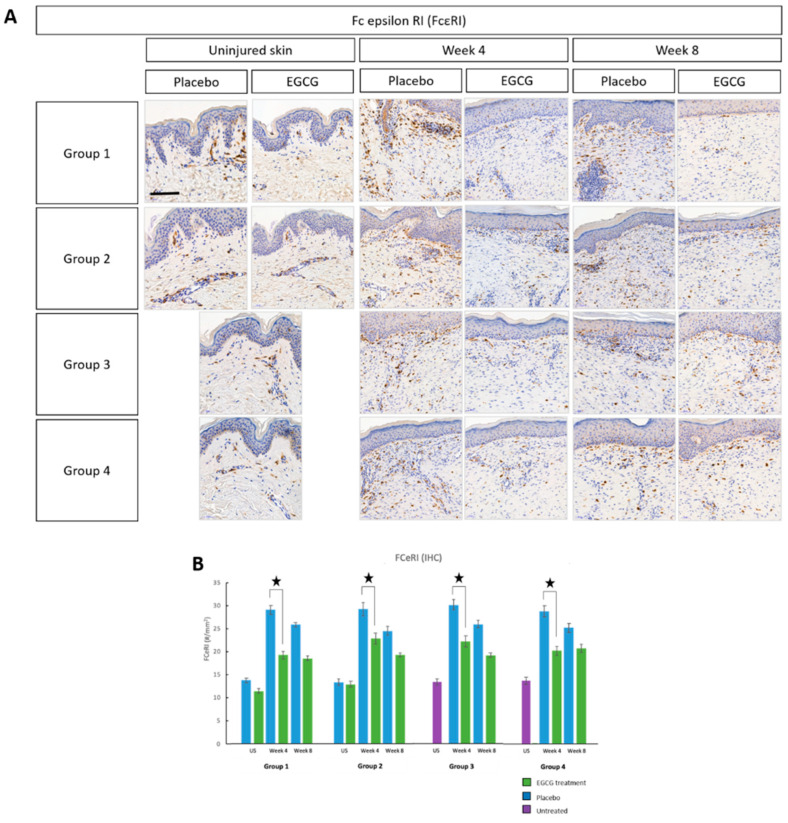
Immune cell marker analysis of topical epigallocatechin-3-gallate (EGCG) versus placebo (Fc epsilon RI (FcεRI)). (**A**) FcεRI immunohistochemical images demonstrated a reduction in expression in EGCG samples compared to placebo. Scale bars = 50 μm. (**B**) There were greater levels of FcεRI in scar tissue compared to uninjured skin predominantly at scar edges and less centrally, and this was highest at week 4 in all groups. There was a significant reduction in FcεRI at week 4 in EGCG treated samples compared to placebo in all groups (*p* < 0.01) but no significant difference between groups. Significance: ★ *p* < 0.05 Scale bars = 50 μm.

**Figure 8:**
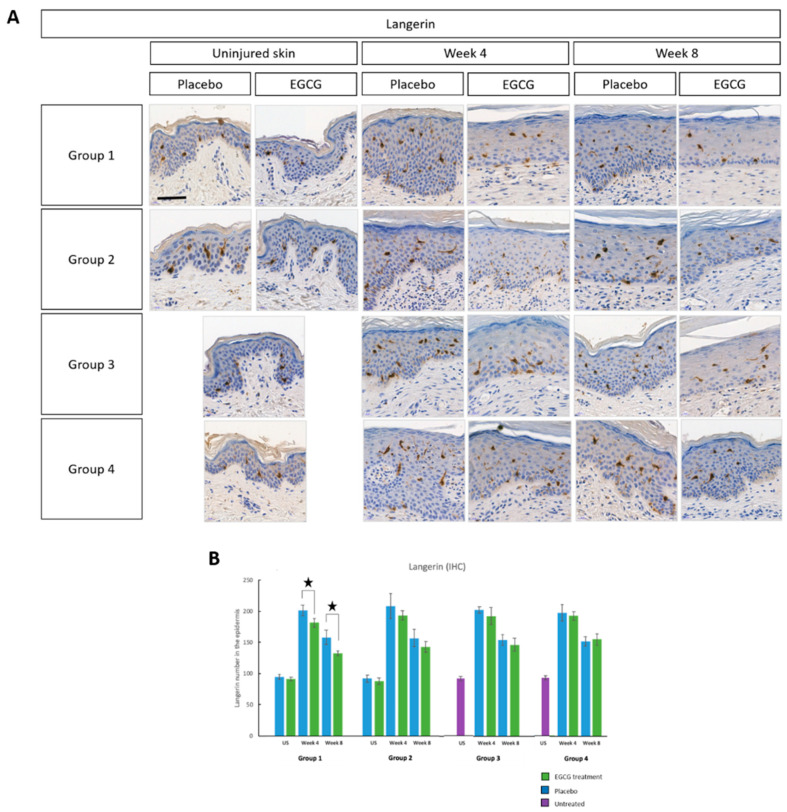
Immune cell marker analysis of topical epigallocatechin-3-gallate (EGCG) versus placebo (Langerin). (**A**) Langerin immunohistochemical images showed reduced expression in EGCG samples compared to placebo samples. Scale bars = 20 μm. (**B**) Analysis of the total number of cells in the whole epidermis demonstrated a significant reduction in EGCG-treated samples compared to placebo samples at week 4 in Group 1 only (*p* = 0.02). No differences were observed between the groups. Significance: ★ *p* < 0.05 Scale bars = 50 μm.

In support of the above, we additionally analyzed several other markers including Fc epsilon RI (FcεRI), langerin, M1 and M2 macrophages and CD8 T-cells. There were greater levels of FcεRI in scar tissue compared to uninjured skin predominantly at scar edges and less centrally, and this was highest at week-4 in all groups (Figure 7). There was a significant reduction in FcεRI at week-4 in EGCG-treated samples compared to placebo in all groups (*p* < 0.01). Langerin analysis demonstrated a significant 13% reduction in EGCG-treated samples compared to placebo samples at week-4 in group-1 only (*p* = 0.02) (Figure 8).

**Figure 9:**
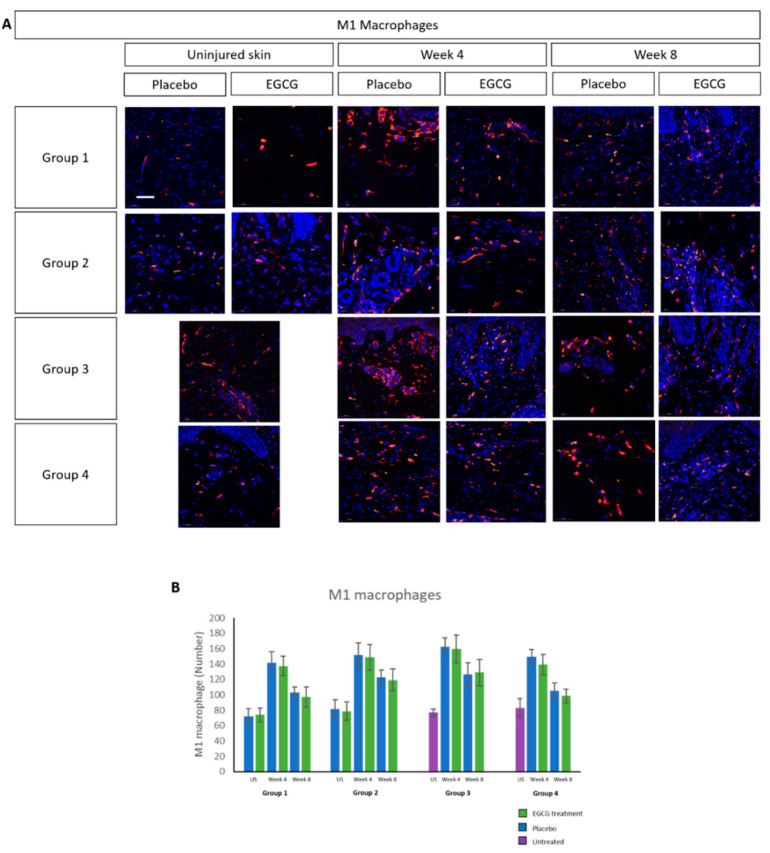
Immune cell analysis of total number of M1 macrophages in whole tissue. (**A**) M1 macrophage analysis demonstrated higher expression at week 4 compared to uninjured skin, reaching levels similar to uninjured skin by week 8 in treated and placebo samples in all groups. (**B**) There were no significant differences seen in EGCG samples compared to placebo samples or between the groups. Scale bars = 50 μm.

**Figure 10:**
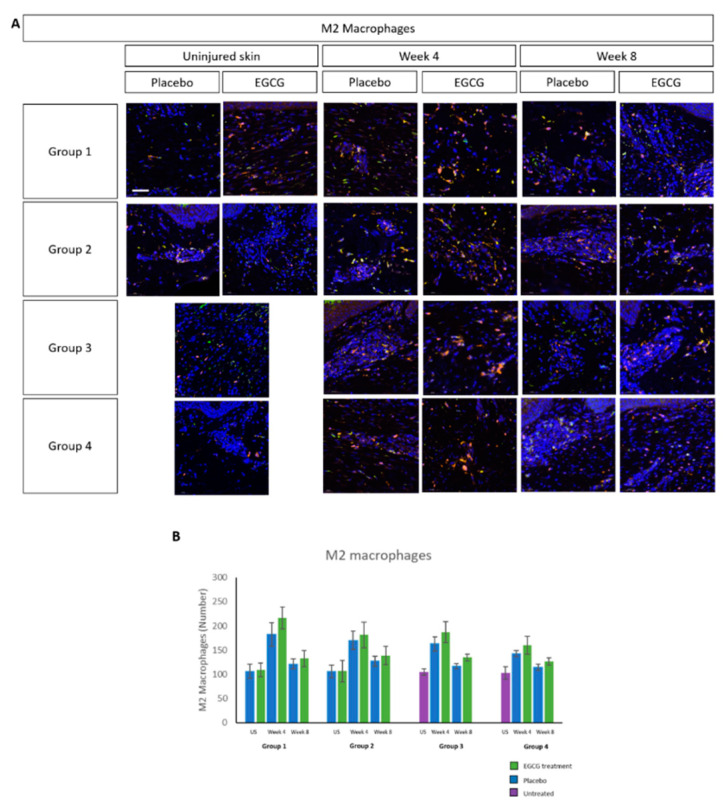
Immune cell analysis of total number of M2 macrophages in whole tissue. (**A**) There was an increase in M2 macrophages in both treated and placebo samples, with greater numbers seen in EGCG-treated scars by week 4 after injury compared with placebo scars. (**B**) Measurements showed that levels were similar when comparing placebo with EGCG treated samples at each time point. Scale bars = 50 μm.

**Figure 11:**
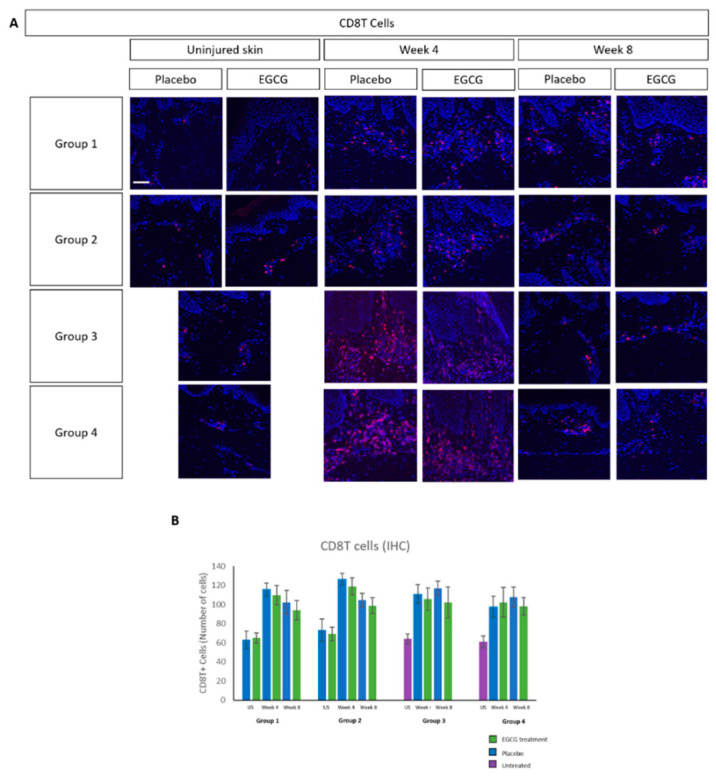
Immune cell analysis of total number of CD8T cells in whole tissue. (**A**) CD8T cell marker analysis showed that levels were higher than uninjured skin in all groups and in placebo and treated samples. (**B**) CD8T cell levels were similar at week 4 and at week 8 and no significant differences were noted between treated and placebo or between groups. Scale bars = 50 μm.

M1 macrophage analysis demonstrated higher expression at week-4 compared to uninjured skin, reaching levels similar to uninjured skin by week-8 in treated- and placebo samples in all groups (Figure 9). There was an increase in M2 macrophages over time in both treated and placebo-samples, although differences were not statistically significant (Figure 10). CD8 T-cell marker analysis showed that levels were higher than uninjured skin in all groups and in placebo- and EGCG-treated samples (Figure 11). Levels were similar at week-4 and at week-8 and no significant differences were noted between treated and placebo or between groups.

### 3.3. Significant Reduction of Blood Flow Is Seen in 7-Day Pre-Injury Priming

**Figure 12:**
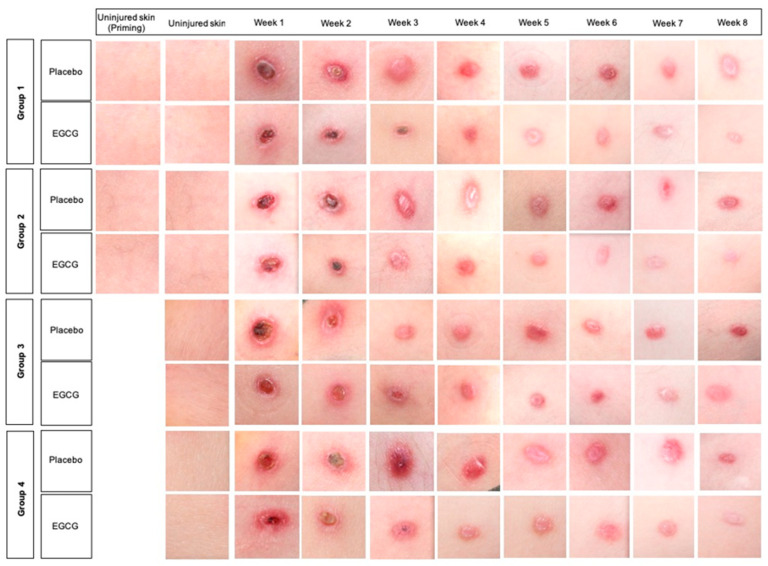
Clinical images of the scar sites over 8 weeks for each of the groups. Scars healed well in all groups and with both topical formulations. Scars were slightly paler in color with EGCG compared to placebo. No great visual differences were noted between the groups.

**Figure 13:**
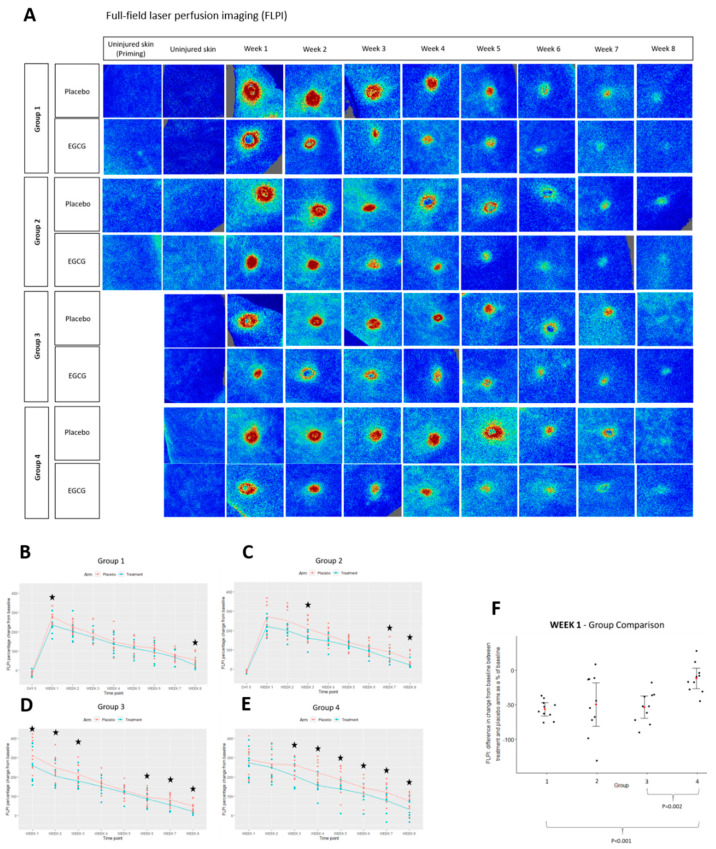
Clinical analysis of blood flow using non-invasive objective device; full-field laser perfusion imaging (FLPI). (**A**) FLPI images of epigallocatechin-3-gallate (EGCG) compared to placebo in all groups. (**B**) Group 1 FLPI measurements. (**C**) Group 2 FLPI measurements. (**D**) Group 3 FLPI measurements. (**E**) Group 4 FLPI measurements. (**F**) Between group comparison analysis for FLPI at week 1. Significance: ★ *p* ≤ 0.01.

Clinical images of skin biopsy scars can be found in Figure 12. We performed initial blood flow analysis by using non-invasive objective measures: Full-field laser perfusion imaging (FLPI) and Dynamic Optical Coherence tomography (D-OCT). FLPI measurements showed that blood flow progressively reduced over 8-weeks from week 1 in all groups and in treated and placebo arms (Figure 13). There was a significant reduction in EGCG arms compared to placebo arms across all groups; group-1 at weeks-1 and 8 (*p* < 0.01), group-2 at weeks-3, 7 and 8 (*p* < 0.01) group-3 at weeks 1–3 and 6–8 (*p* ≤ 0.01) and group-4 was significantly reduced at weeks 3–8 (*p* < 0.01). Between group comparison analysis showed that groups-1 and 3 reduced blood flow more than group-4 at week-1 (*p* < 0.001, *p* = 0.002, respectively).

**Figure 14:**
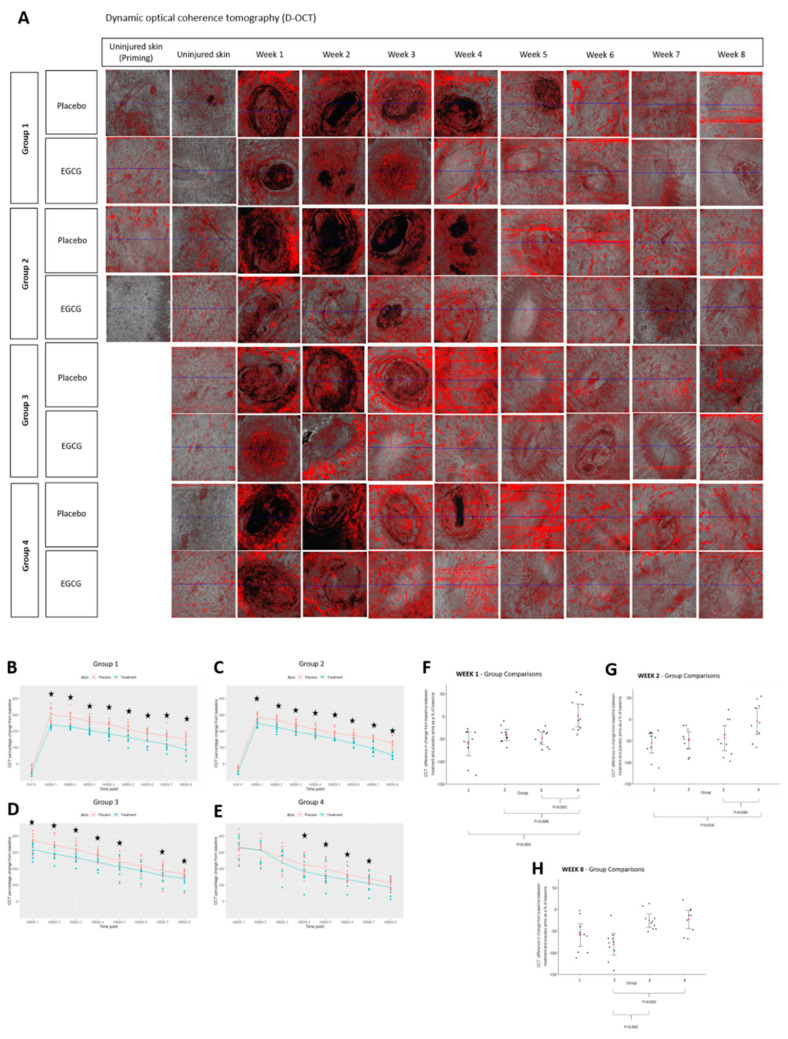
Clinical analysis of blood flow using non-invasive objective device; dynamic-optical coherence tomography (D-OCT). (**A**) D-OCT images of EGCG arms compared to placebo. (**B**) Group 1 D-OCT measurements. (**C**) Group 2 D-OCT measurements. (**D**) Group 3 D-OCT measurements. (**E**) Group 4 D-OCT measurements. (**F**) Group comparison analysis for D-OCT at week 1. (**G**) Group comparison analysis for D-OCT at week 2. (**H**) Group comparison analysis for D-OCT at week 8. Significance: ★ *p* ≤ 0.01.

D-OCT analysis of blood flow also demonstrated a reduction over time from week 1 in both arms and in all groups (Figure 14). This was significantly decreased in the EGCG-treated compared to the placebo; group-1 at weeks 1–8 (*p* < 0.01) and group-2 at weeks 1–8 (*p* < 0.01), group-3 at weeks 1–5, 7 and 8 (*p* < 0.01) and group-4 at weeks 4–7 (*p* < 0.01). Group comparison analysis demonstrated that groups 1–3 reduced blood flow more than group-4 at week-1 (*p* < 0.001, *p* = 0.006, *p* = 0.001, respectively). Priming Group-1 had a greater difference than group-4 at week-2 (*p* = 0.016), whilst group-2 reduced more than groups-3 and -4 at week-8 (*p* = 0.003, *p* = 0.002, respectively).

### 3.4. Angiogenic Markers Are Most Downregulated in Pre-Injury Priming 

**Figure 15:**
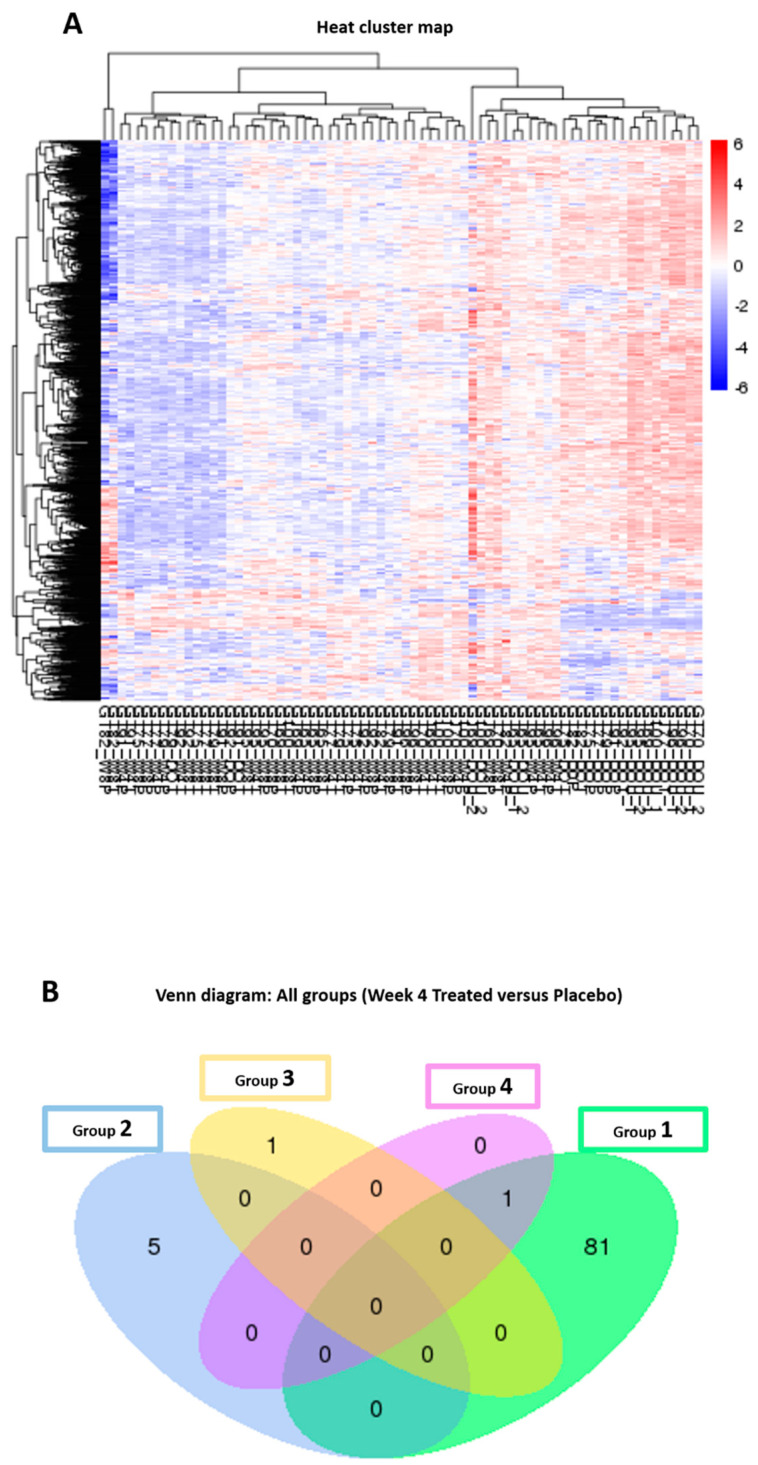
mRNA-sequencing results (Cluster analysis and Venn diagram). (**A**) Cluster analysis of differential expression genes. Hierarchical clustering analysis was carried out with the log_10_(FPKM + 1) of union differential expression genes of all comparison groups under different experimental conditions. Highly expressed genes are indicated in red, and genes with low expression are indicated in blue. Samples were ordered from those with lowest expression levels on the left to highest expression levels on the right side. (**B**) Venn diagrams to illustrate the number of common and unique differential expression genes among comparison groups. Group 1 had the greatest number of differentially expressed genes compared to all other groups.

**Figure 16:**
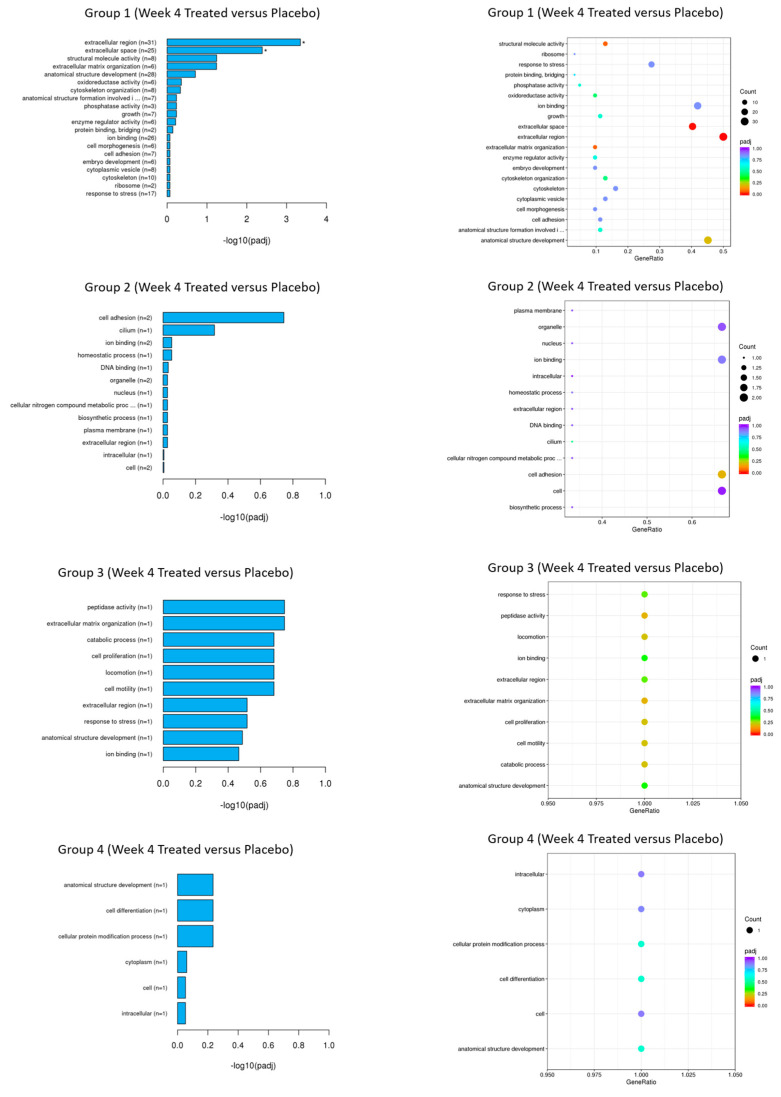
mRNA-sequencing results (Gene Ontology). Gene Ontology. The Gene Ontology (GO) enrichment analysis results are displayed through bar and dot plots. They display the number of genes that are significantly enriched in each GO term.

mRNA sequencing analysis demonstrated in group-1, the most significantly differentially expressed genes which were reduced with EGCG compared to placebo were hemoglobin subunit beta (HBB), hemoglobin subunit alpha-1 (HBA1) and hemoglobin subunit alpha-2 (HBA2) at week-4 (Figure 15 and Figure 16, Table 6).

**Figure 17:**
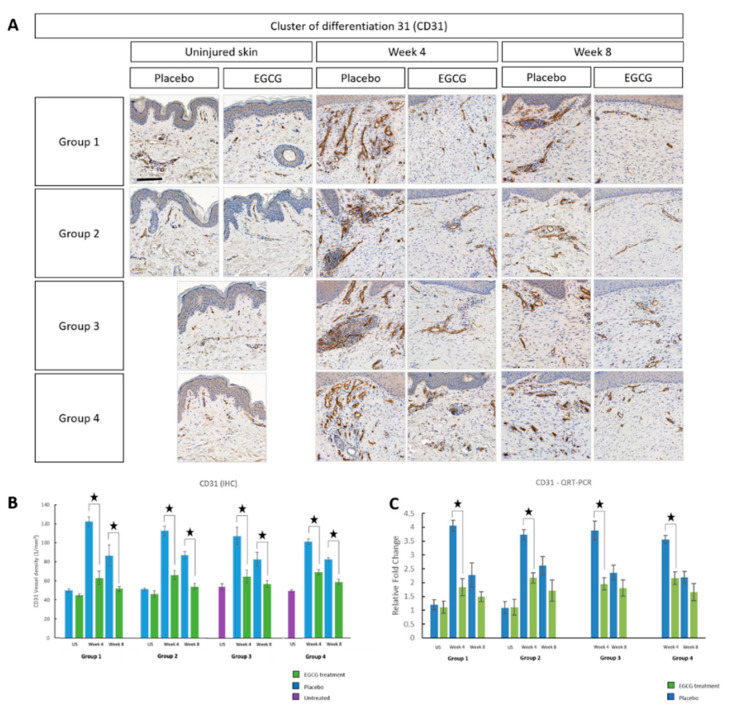
Angiogenesis marker CD31 analysis of topical epigallocatechin-3-gallate (EGCG) versus placebo. (**A**) Cluster of differentiation 31 (CD31) images demonstrated a reduction in vessel density in EGCG samples compare to placebo. (**B**) CD31 vessel density measurements. (**C**) Quantitative real-time reverse transcriptase–PCR (QRT-PCR) analysis for CD31. Significance: ★ *p* ≤ 0.01. Error bars: mean ± SD. Scale bars = 50 μm.

In order to further support these findings and the clinical findings, we used two well-known and established angiogenic immunohistochemical markers; CD31 and VEGF-A. CD31 was significantly down-regulated in EGCG-treated samples compared with placebo at weeks-4 and -8 in all groups (*p* < 0.01) (Figure 17). There was a significant difference between groups (*p* = 0.02) at week-4. Group 1 was shown to have the largest reduction in CD31 compared to group-4 by 40% (*p* = 0.01). QRT-PCR for CD31 demonstrated significant down regulation at week-4 in all groups in EGCG-treated samples compared to placebo samples.

**Figure 18:**
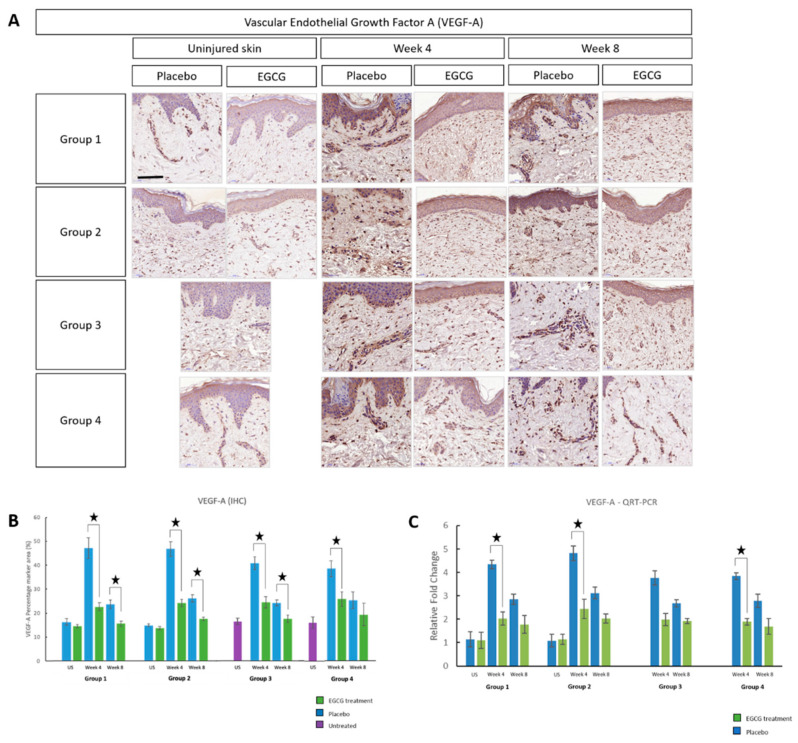
Angiogenesis marker Vascular Endothelial Growth Factor A (VEGF-A) analysis of topical epigallocatechin-3-gallate (EGCG) versus placebo. (**A**) VEGF-A images also confirmed a decrease in EGCG samples compared to placebo. (**B**) VEGF-A (epidermal and dermal) percentage marker area measurements. (**C**) QRT-PCR of VEGF-A. Significance: ★ *p* ≤ 0.01. Error bars: mean ± SD. Scale bars = 50 μm.

To further corroborate these findings, VEGF-A was shown to be significantly down-regulated at week-4 in all groups in EGCG-treated samples compared to placebo (*p* < 0.01) (Figure 18). Furthermore, groups 1–3 showed that there was a significant reduction at week-8 in EGCG-treated samples (*p* < 0.01). Between group analysis demonstrated a significant difference (*p* = 0.003) at week-4. This difference was found to be that group-1–2 had significantly greater reductions compared to group-4 (51%: *p* = 0.005, 56%: *p* = 0.01, respectively). Gene expression analysis of VEGF-A displayed significant reductions in EGCG-treated samples compared to placebo at week-4 (*p* < 0.05) in groups 1–2 and 4, although there were no significant differences between groups.

### 3.5. Antioxidant Effects Are Enhanced Following Anti-Scarring Topical Application 

**Figure 19:**
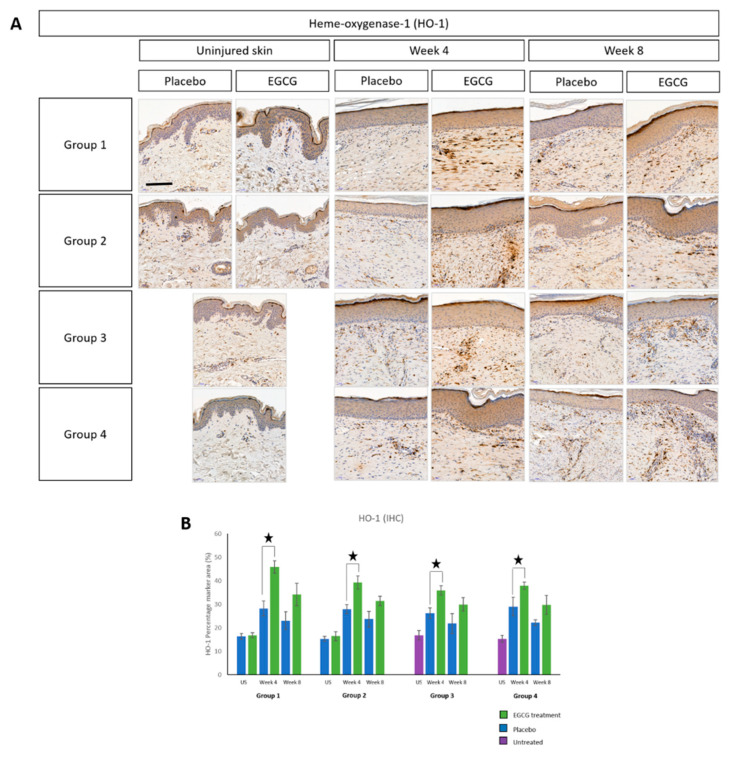
Antioxidant Heme-oxygenase 1 (HO-1) effects of topical epigallocatechin-3-gallate (EGCG) versus placebo. (**A**) HO-1 immunohistochemical stain images. (**B**) HO-1 percentage marker area measurements. Scale bars = 100 μm. Error bars: mean ± SD. Significance: ★ *p* ≤ 0.01.

**Figure 20:**
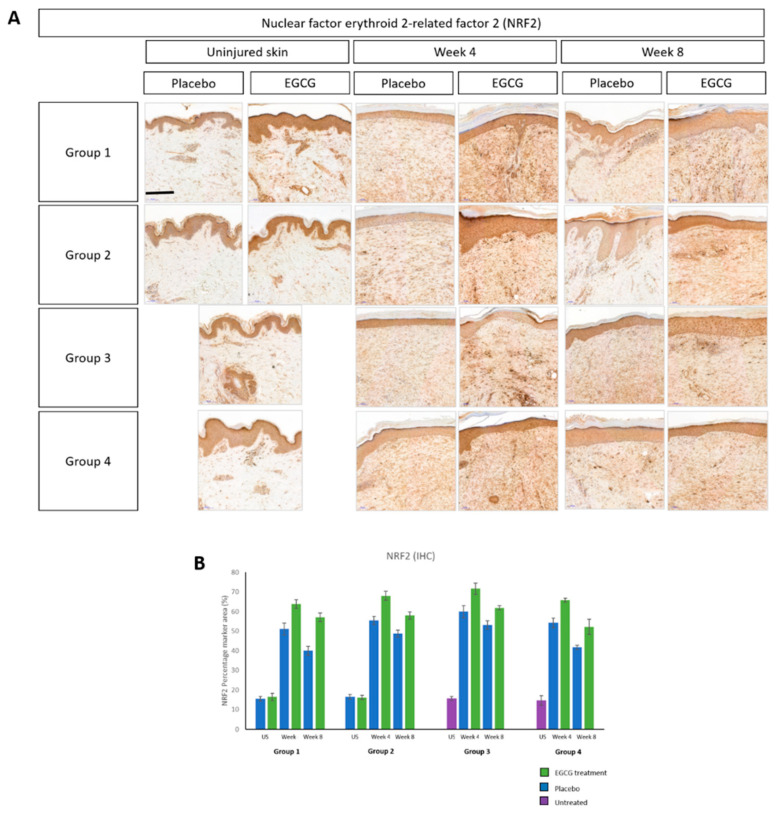
Antioxidant Nuclear factor erythroid 2-related factor 2 (NRF2) effects of topical epigallocatechin-3-gallate (EGCG) versus placebo. (**A**) NRF2 immunohistochemical stain images. (**B**) NRF2 percentage marker area measurements. Scale bars = 100 μm. Error bars: mean ± SD. Significance: ★ *p* ≤ 0.01.

As previously reported, EGCG has been found to have an antioxidant effect. Therefore, we used Hemeoxygenase-1 (HO-1) (Figure 19) and Nuclear factor erythroid 2-related factor 2 (NRF2) (Figure 20) as immunohistochemical markers to identify any changes in our scar samples. HO-1 was found to be higher in scar tissue compared to uninjured skin at weeks-4 and 8 in all groups. EGCG-treated samples showed higher levels of HO-1 compared to placebo samples in all groups. This was significantly higher at week-4 in all groups (*p* < 0.01), although no significant differences were found between the groups. NRF2 analysis demonstrated the same trend where levels were greatest at week-4 and 8 compared to uninjured skin. EGCG samples had slightly higher amounts of NRF2 compared to placebo samples, although not significantly.

### 3.6. Scar Thickness Is Significantly Reduced with Anti-Scarring Topical Application

**Figure 21:**
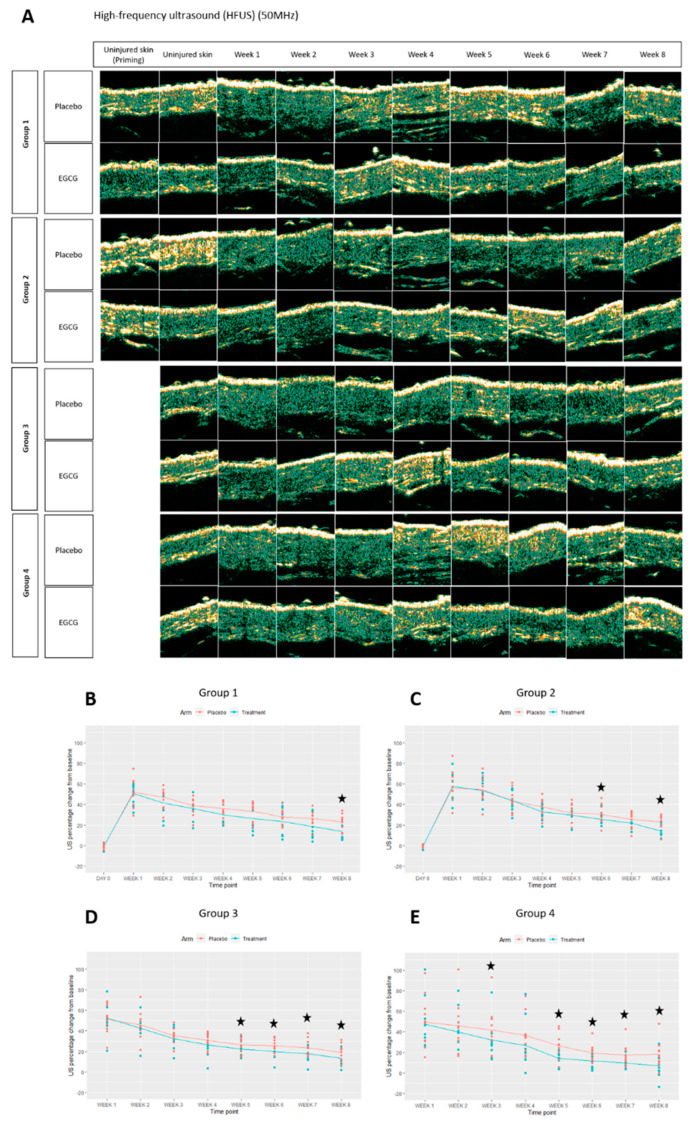
Skin thickness analysis (High frequency ultrasound (HFUS)) of topical epigallocatechin-3-gallate (EGCG) versus placebo. (**A**) Images of skin/scar thickness using high frequency ultrasound (HFUS). (**B**) Group 1 skin thickness HFUS. (**C**) Group 2 skin thickness HFUS. (**D**) Group 3 skin thickness HFUS. (**E**) Group 4 skin thickness HFUS. Significance: ★ *p* ≤ 0.01. Error bars: mean ± SD. Scale bars = 100 μm.

**Figure 22:**
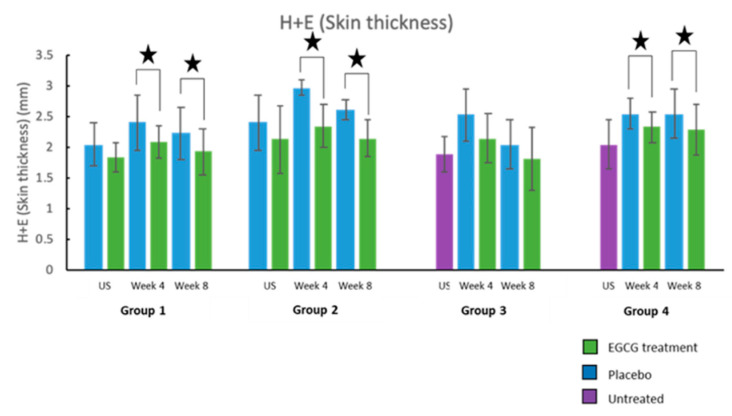
H + E skin/scar thickness. H + E scar thickness measurements demonstrated reduced scar thickness with EGCG at weeks 4 and 8 in Group 1 (*p* = 0.03, *p* = 0.03, respectively), Group 2 (*p* = 0.04, *p* = 0.002, respectively) and Group 4 (*p* = 0.01, *p* = 0.03, respectively). Significance: ★ *p* < 0.05.

Structural changes in the scars were assessed clinically by high frequency ultrasound (HFUS) and elasticity probe, and elastin and collagen markers. HFUS was used to measure scar thickness clinically at every time point over 8 weeks. Scar thickness measurements were found to be lower in EGCG scars compared to placebo scars across the groups (Figure 21). This was significant in group-1 at week-8 (*p* = 0.002), group-2 at week-6 and 8 (*p* < 0.01) group-3 at weeks 5–8 (*p* < 0.01) and group-4 at weeks 3, 5–8 (*p* < 0.01). H + E scar thickness measurements also corroborated these findings and showed reduced scar thickness with EGCG at weeks 4 and 8 in Groups 1,2 and 4 (Figure 22).

**Figure 23:**
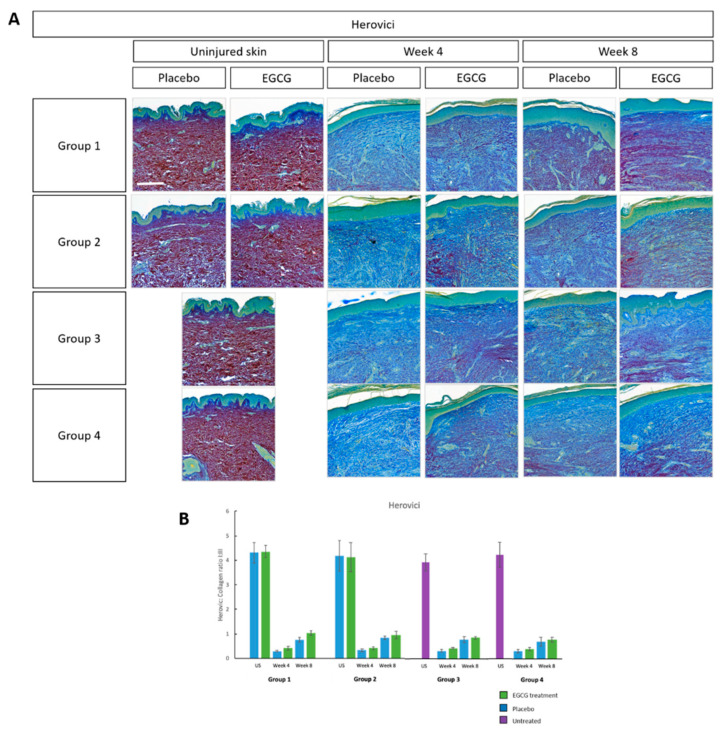
Herovici analysis of topical epigallocatechin-3-gallate (EGCG) versus placebo. (**A**) Herovici images demonstrating mature and immature collagen. (**B**) Herovici collagen I:III ratio analysis. Error bars: mean ± SD. Scale bars = 100 μm.

Herovici collagen I:III ratio analysis was performed to establish any changes in collagen levels between the EGCG- and placebo-samples (Figure 23). All groups demonstrated the greatest ratio in uninjured skin compared to week-4 and 8 samples. There was a slightly greater ratio in week-8 samples compared to week-4 samples. No significant differences between EGCG-treated and placebo-samples were noted in any group or between groups.

### 3.7. Elastin Content Is Most Upregulated in Pre-Injury Priming

**Figure 24:**
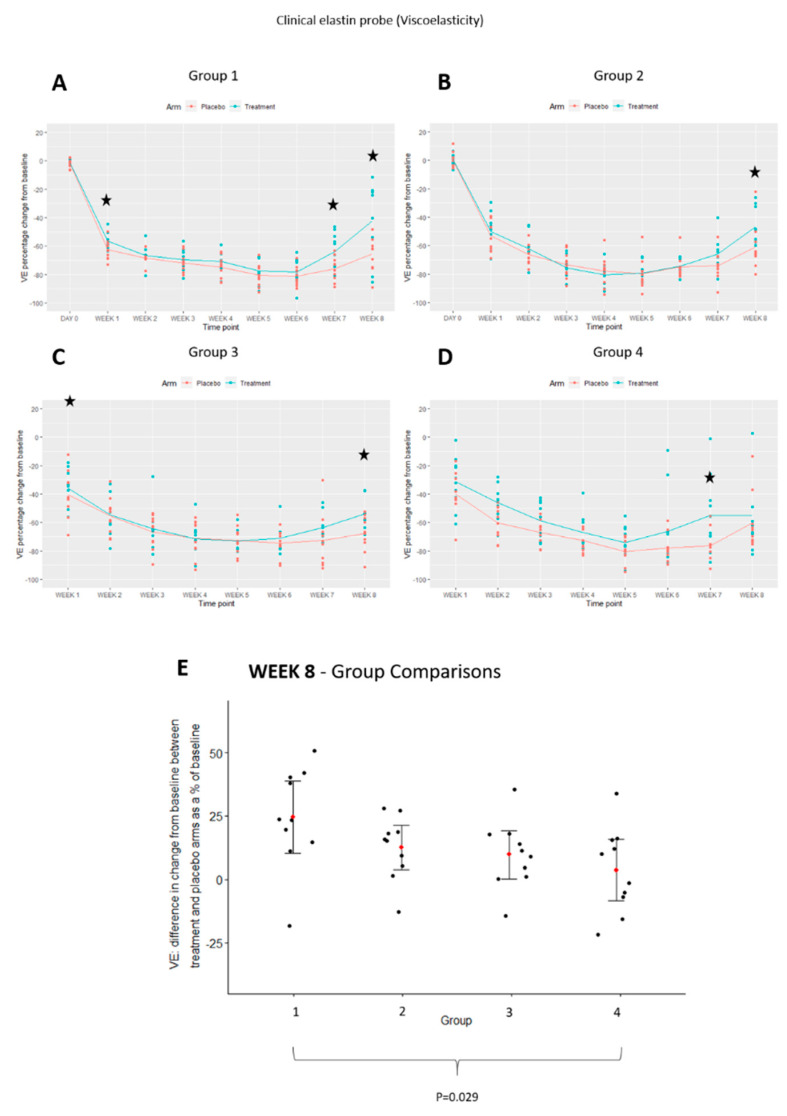
Clinical elastin probe analysis of topical epigallocatechin-3-gallate (EGCG) versus placebo. (**A**) Clinical elastin probe viscoelasticity measurements are presented for Group 1. (**B**) Group 2 viscoelasticity measurements. (**C**) Group 3 viscoelasticity measurements. (**D**) Group 4 viscoelasticity measurements. (**E**) Between group comparisons for viscoelasticity at week 8. Significance: ★ *p* ≤ 0.01.

A clinical elastin probe was used to measure the viscoelasticity of the scars over 8-weeks (Figure 24). Elasticity was found to be increased in EGCG-treated arms compared to placebo-treated arms predominantly at later time points in all groups. This was significantly increased with EGCG-treated in group-1 at weeks-1, 7 and 8 (*p* = 0.008, *p* < 0.001, *p* = 0.006, respectively), group-2 at week-8 (*p* = 0.005), group-3 at week-8 (*p* = 0.003) and group-4 at week-7 (*p* = 0.012). Between group comparisons showed that group-1 significantly increased elastin more than group-4 at week-8 (*p* = 0.029).

**Figure 25:**
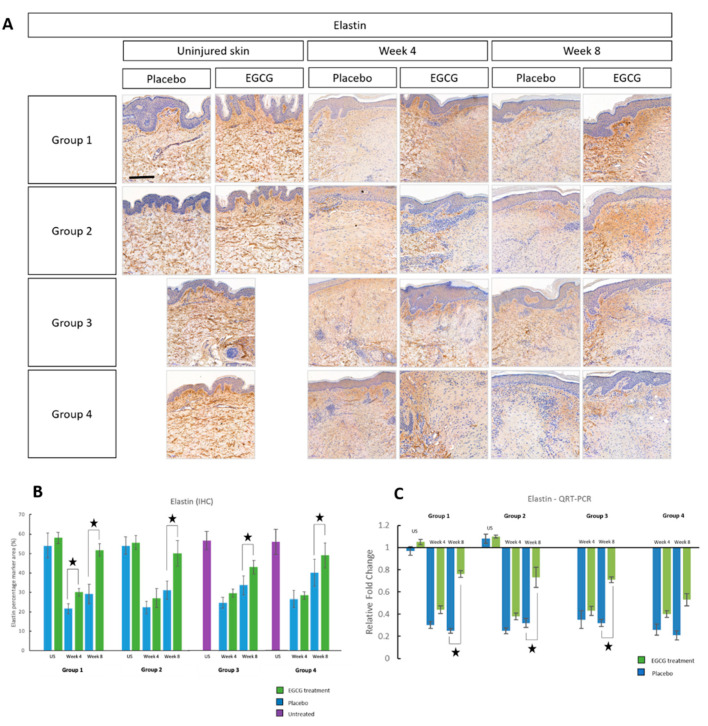
Immunohistochemical analysis of elastin for topical epigallocatechin-3-gallate (EGCG) versus placebo. (**A**) Immunohistochemical images for elastin. (**B**) Elastin percentage marker area measurements. (**C**) Quantitative real-time reverse transcriptase–PCR (QRT-PCR) analysis for elastin. Significance: ★ *p* ≤ 0.01. Error bars: mean ± SD. Scale bars = 100 μm.

Immunohistochemical analysis of elastin was up-regulated at week-8 in EGCG-treated samples compared to placebo samples in all groups (*p* < 0.01) (Figure 25). There was also a significant increase at week-4 in group-1 only by 12% with EGCG (*p* = 0.01). Between group analysis indicated a significant difference at week-8 (*p* = 0.001). The greatest differences were in group-1 compared to groups-3 by 20% (*p* = 0.008) and group-4 by 20% (*p* = 0.003) and in group-2 compared to group-4 by 17% (*p* = 0.009). Gene expression analysis of elastin demonstrated that there was an upregulation in EGCG-treated samples compared to placebo samples at week-4 and 8, and this was significant in groups-1 compared to 3 at week-8 (*p* < 0.05).

## 4. Discussion

**Figure 26:**
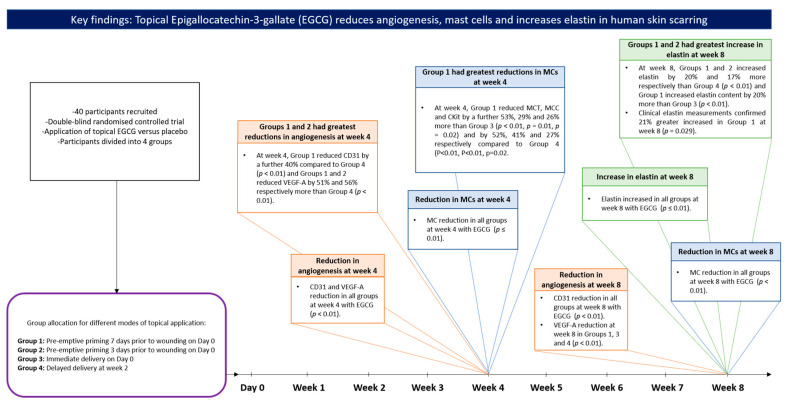
Key findings: Topical Epigallocatechin-3-gallate (EGCG) reduces angiogenesis, mast cells and increases elastin content in human skin scarring. A summary of the methodology of the study and the key outcomes linked with the corresponding time points.

This double-blind, randomized placebo-controlled clinical trial quantitatively investigates the effects of different timings of application of topical anti-scarring formulation (EGCG) versus a placebo in relation to inflammatory response, angiogenesis, antioxidant effects and structural changes in cutaneous skin scarring in healthy human volunteers (Figure 26).

Our findings demonstrated that EGCG topical application: (1) Mast cell (MCT, MCC and CKit) number was significantly reduced, (2) Blood flow and angiogenesis (CD31 and VEGF-A expression) were significantly reduced, (3) Antioxidant effect was enhanced by increased HO-1 levels, (4) Scar thickness was reduced, (5) Viscoelasticity increased and elastin expression was significantly increased.

The unique concept of priming the skin with topical EGCG prior to wounding in human skin scarring has, to our knowledge, not yet been fully investigated prior to this study. Our results demonstrated that pre-emptive pre-injury priming groups by 7-days (group-1) or 3-days (group-2) were superior in comparison to immediate (group-3) or delayed topical application groups (group-4). Based on immunohistochemical data, mast cell analysis by MCT, MCC and CKit demonstrated that at week-4, Group-1 showed greater reductions than group-3 (53%, 29%, 26%, respectively) and group-4 (52%, 41%, 27%, respectively), whilst group-2 showed greater reductions in MCC compared to group-4 (29%). Angiogenesis analysis by CD31 and VEGF-A showed that group-1 was optimal compared to group-4 (40%, 51%, respectively) at week-4 and group-2 reduced VEGF-A levels more than group-4 (56%). Elastin content was significantly increased in groups-1 and 2 compared to group-4 (20%, 17%, respectively) at week-8 and this was supported by clinical elastin measurements in group-1 at week-8. There were also differences noted in the primed uninjured skin prior to injury indicating that by applying EGCG this reduces the resident mast cell population, reduces angiogenic markers and increases elastin levels initially prior to the effects becoming more maximized when the injury occurs and with further topical application (Table 7).

Several studies support the concept of pre-emptive priming of skin. For instance, research on the treatment of pigmented acne scars by ablative laser therapy advocates the use of priming agents to reduce wound healing time, decrease the risk of post-inflammatory hyperpigmentation and provide ultraviolet damage protection [10]. Resurfacing of scars using fractional CO2 laser with early interventional treatment can reduce scar formation [11]. Prophylactic negative pressure wound therapy promotes surgical wound healing and reduces surgical site infections [12,13]. A murine study showed beneficial effects with pre-treatment by pro-angiogenic growth factors in the healing of diabetic incisional wound [14]. Of note, depigmenting agents such as hydroquinone or retinoic acid are indicated 2–4 weeks prior to undergoing chemical peels [15].

Continuous inflammation can stimulate the secretion of pro-inflammatory cytokines which can lead to excessive scarring such as hypertrophic and keloid scar formation [28]. We showed that EGCG had an inhibitory effect on several key markers including langerin, FcεRI and mast cell markers. Epidermal Langerhans cells are a component of the skin’s defense system and evidence suggests that these cells play a role in the initiation and resolution of wound healing particularly during excessive scarring [29]. It has been shown that human hypertrophic scars display higher numbers of CD1a+ Langerhans cells compared with normal scars [29]. Additionally, our group have previously demonstrated differences in the immune cell population between normal and keloid scars and showed that there were no significant differences in Langerhans cell count between the two types of scars [30]. In support of our findings, we also showed inhibition of FcεRI which is an essential component of allergic reaction, has been shown to be expressed on normal epidermal Langerhans cells [31] and is responsible for inducing mast cell degranulation [32,33]. Mast cells affect fibroblasts present in the remodeling phase of healing, and thus affect regulation of scar formation [28]. Indeed, mast cell expression increases in normotrophic and hypertrophic skin scars compared to normal skin and this increases with scarring severity [30]. Blocking mast cell function could therefore be used to minimize or prevent excessive scarring [34,35,36].

Our group have previously shown that EGCG targets MCT and MCC in both hypertrophic and keloid plus normal scars in ex vivo human skin scar models [26,27]. In addition, our previous double-blind randomized placebo-controlled trial in healthy human volunteers also corroborated the above results by showing a reduction in mast cells post-EGCG treatment [16]. Interestingly, using topical EGCG to treat psoriasis-like inflammation of BALB/c mice demonstrated a similar anti-inflammatory effect [37]. Another murine study used a chitosan-based polymeric nanoparticle formulation for topical EGCG delivery for treating psoriasis and showed reductions in angiogenesis and immune cells including mast cells [38]. EGCG is an inhibitor of inflammation and macrophage accumulation in the treatment of diabetic wound healing in mice [13]. Shin et al., 2007 [39] demonstrated that EGCG regulated the allergic inflammatory response in the human mast cell line.

Angiogenesis, which is the generation of new blood vessels, is an important aspect of wound healing [40]. The new blood vessels are required in order to deliver oxygen and nutrients to the wound site. Many studies have shown that VEGF is a key proangiogenic mediator in wound healing [41]. VEGF contributes to the formation of excessive angiogenesis in skin scarring [42,43,44,45,46]. EGCG, however reduces angiogenesis in human skin where HIF-1α and VEGF expression were inhibited [47], and in a study by Zhang et al., 2006 [48] in human cervical carcinoma and hepatoma cells, EGCG downregulated HIF-1α and VEGF expression. EGCG is a potent inhibitor of IL-8 release by TNF alpha-stimulated normal human keratinocytes and downregulates VEGF and IL-8 [49]. Thus, partial inhibition of angiogenesis with EGCG is likely favorable in order to prevent excessive scar formation.

Antioxidant mechanisms can protect cells from reactive oxygen species induced damage [50]. However, if the balance is disturbed the tissue will undergo oxidative stress which can delay wound healing [50,51]. Oxidative stress is an imbalance of oxygen, nitrogen-based free radical production, cellular antioxidant defense system [52] and important in fibrosis [53]. Antioxidants protect cells from the environment. Our findings demonstrated an increase in HO-1 staining, suggesting a possible enhanced antioxidant effect with EGCG on scarring. Previous studies including Kim and Lee 2016 [54] found that EGCG inhibited inflammatory responses by suppressing the production of proinflammatory cytokines through HO-1 induction during adipocyte–macrophage interaction. Additionally, NRF2-mediated HO-1 overexpression confers resistance to apoptosis induction by EGCG [55]. Furthermore, EGCG blocked UVB-induced infiltration of leukocytes and generation of ROS in human skin [56]. Zhu et al., 2014 [57] demonstrated EGCG induced the expression of HO-1 via transcriptional activation. HO-1 knockdown or treatment with an HO-1 inhibitor that reversed the protective role of EGCG. EGCG has preventive effect against radical-evoked apoptosis by downregulation of caspase-8 and -3 in HaCaT cells [58]. EGCG exhibits antioxidant properties by increasing the expression of HO-1 [59]. Further tests are required in order to elucidate the antioxidant effects of this topical formulation on skin scarring.

Scar assessment should involve the objective quantitative evaluation of a number of key parameters [60] including inflammation and angiogenesis as well as anatomical structural features specifically, thickness and elasticity of the scar tissue. Elastic fibers have been shown to play an important role in the skin’s structure and function [61]. Changes in the content, quality and organization of elastin have been implicated in the pathogenesis of scar formation [62]. It has been demonstrated in hypertrophic and keloid scars that there are alterations in the quality and quantity of elastin and collagen [62]. In a study by Roten et al., elastin fibers were examined in newly acquired human excised scars and they demonstrated that 91% of these scars were lacking elastin for 3 months and subsequently they produced new elastin in 40% of the scars [61]. We showed that EGCG reduced scar thickness and increased elastin content. A previous study by Chiu et al., 2005 [63] confirmed increased elastin content with EGCG. Syed et al., 2013 [26] studied effects of EGCG on keloid tissue and showed induced epidermal shrinkage, reduced collagen-I and -III and keloid volume. Another study reported reductions in mouse ear and skin thickness following EGCG in a murine psoriasis-like dermatitis model [38].

The exact mechanism of action of EGCG for the treatment of skin scarring has yet to be fully established. There are promising findings in relation to the inhibition of mast cell density, inhibition of angiogenesis, down regulation of profibrotic pathways and increased antioxidant effects. Inhibition of inflammatory responses is suggested to be due to the suppression of the activation of PI3K/Akt/STAT3 pathways [64]. Whilst the suppression of profibrotic pathways including PI3K, STAT3, MMP2, MMP9, PDGF may lead to scar reduction. Phosphorylation of p38 MAPK is inhibited by EGCG [65]. Catechins inhibit MC-stimulated type I collagen expression by suppressing activation of the PI3k/Akt/mTOR signaling pathways in keloid fibroblasts [48]. Furthermore, EGCG antioxidant effects likely due to increased activation of NF-E2-related factor and NF-kbeta and thus higher expression of antioxidant markers including HO-1 [66].

This study has demonstrated promise as a unique approach in improving skin scarring outcome. However, future work is necessary to further elucidate these findings and identify if these effects are similar in other skin types, differ between genders and repeat using larger cohorts in different scar endotypes [67]. Overall, effective scar therapies and robust clinical trials to demonstrate the efficacy of scar therapies are lacking, without clear definitions of criteria for scar improvement, along with the heterogenous nature of scar endotypes, leading to difficulties in interpretation and management guideline implementation.

In conclusion, we have demonstrated that pre-emptive pre-injury priming of skin, prior to surgically induced wounds, with topical anti-scarring formulation (EGCG) has a potential beneficial role on human cutaneous skin scarring by reducing mast cells, angiogenesis and simultaneously increasing elastin content.

## Figures and Tables

**Table 1:** Demographic data. Demographic information for 40 healthy volunteers.

Demographics	Characteristics	Number	Percentage (%)
Participants	Total	40	100
Gender	Male	15	37.5
	Female	25	62.5
Ethnicity	Caucasian	38	95
	Other	2	5
Age (years)	21–30	23	57.5
	31–35	17	42.5

**Table 2:** Objective non-invasive device modalities which were used at each weekly time point over 8 weeks during this study to monitor the progression of healing.

Objective Non-Invasive Device	Description
Full-field Laser Perfusion Imaging (FLPI) (Moor Instruments Ltd., Axminster, UK)	- Measures blood flow in the skin’s microcirculation - Tissue thickness sampled is 1 mm - Capillary diameters up to 10 μm - Flow rates of 0.01–10 mm/s
Dynamic-optical coherence tomography (D-OCT) (Vivosight, Michaelson, UK)	- Detects high-speed changes in back-scattered light caused by moving cells in vessels and produces blood flow measurements by depth - Uses low-coherence near infrared light - Lateral optical resolution of <7.5 μm - Axial resolution of 10 μm - Penetration depth approximately 1.2–1.8 mm - Scan area of 6 mm × 6 mm
High-frequency ultrasound (HFUS) (DermaScan-C, Cortex Technologies, Denmark)	- Uses sound waves and provides measure of skin thickness - Ultrasonic waves partially reflected at the boundary between adjacent structures and produce echoes of different amplitudes - 50 MHz probe - Resolution of 30 × 60 μm - Penetration depth of 3 mm - Scan area of 2.7 × 6 mm
Elastin Probe (Dermalab) (Dermalab, Cortex Technologies, Denmark)	- Principle: Stress/strain by preset vacuum - Measures ViscoElasticity - Probe: 10 mm suction aperture - Low weight (approx. 7 g) for minimum skin bias.

**Table 3:** Immunohistochemical antibodies. Primary antibodies, secondary antibodies, concentration of antibodies, incubation times and detection methods used for immunohistochemistry in this study.

Primary Antibody Name, Product Code and Company	Primary Antibody Raised Species, Isotype and Concentration	Primary ANTIBODY Incubation Details	Secondary Antibody, Company, Concentration, Incubation Details	Detection Method
Mast cell tryptase Ab2378, Abcam, Cambridge, UK	Mouse (monoclonal), IgG1, 1:2000 dilution	Overnight, 4 °C	Alexa Fluor^®^ 647 Goat anti-mouse IgG 1:200 Abcam, ab150119 (30 min room temp)	Fluorescence
Mast cell chymase Ab186417, Abcam, Cambridge, UK	Rabbit (monoclonal), IgG, 1:1000 dilution	Overnight, 4 °C	Alexa Fluor^®^ 488 Goat anti-rabbit IgG 1:200 Abcam, A-11034 (30 min room temp)	Fluorescence
CKIT (CD117) A4502, Dako, UK	Rabbit (polyclonal) IgG, 1:200 dilution	Overnight, 4 °C	Alexa Fluor^®^ 546 Goat anti-rabbit IgG 1:200 Abcam, A-11010 (30 min room temp)	Fluorescence
Anti-Fc epsilon RI Ab54411, Abcam, Cambridge, UK	Mouse (monoclonal) IgG2b, 1:100 dilution	Overnight, 4 °C	Universal antibody by Novolink ^TM^ Leica Biosystems Newcastle ltd, Newcastle Upon Tyne, UK cat. RE7150-K (1 h room temp)	Peroxidase
Langerin Ab192027, Abcam, Cambridge, UK	Rabbit (monoclonal) IgG, 1:1000 dilution	1 h, room temperature	Universal antibody by Novolink ^TM^ Leica Biosystems Newcastle ltd, Newcastle Upon Tyne, UK cat. RE7150-K (1 h room temp)	Peroxidase
CD68 (For M1/M2) M0718, Dako, UK	Mouse (monoclonal) EBM11, 1:50 dilution	Overnight, 4 °C	Alexa Fluor™ 647 Goat anti-mouse IgG 1:200 Abcam, ab150119 (45 min room temp)	Fluorescence
HLA DR + DP + DQ (MHC Class II) (For M1) Ab7856, Abcam, Cambridge, UK	Mouse (monoclonal) IgG1, 1:800 dilution	Overnight, 4 °C	Alexa Fluor™ 546 Goat anti-mouse IgG 1:400 Invitrogen, A11010 (45 min room temp)	Fluorescence
CD206 (For M2) Ab64693, Abcam, Cambridge, UK	Rabbit (polyclonal) IgG, 1:1000 dilution	Overnight, 4 °C	Alexa Fluor™ 488 Goat anti-mouse IgG 1:400 Abcam, A-11034 (45 min room temp)	Fluorescence
VEGF-A, Ab1316, Abcam, Cambridge, UK	Mouse (monoclonal), IgG1, 1:100 dilution	Overnight, 4 °C	Universal antibody by Novolink ^TM^ Leica Biosystems Newcastle ltd, Newcastle Upon Tyne, UK cat. RE7150-K (1 h room temp)	Peroxidase
CD31, Ab134168, Abcam, Cambridge, UK	Rabbit (monoclonal), IgG, 1:300 dilution	1 h, room temperature	Universal antibody by Novolink ^TM^ Leica Biosystems Newcastle ltd, Newcastle Upon Tyne, UK cat. RE7150-K (1 h room temp)	Peroxidase
Heme-oxygenase 1 Ab13248, Abcam, Cambridge, UK	Mouse (monoclonal), IgG1, 1:1000 dilution	Overnight, 4 °C	Universal antibody by Novolink ^TM^ Leica Biosystems Newcastle ltd, Newcastle Upon Tyne, UK cat. RE7150-K (1 h room temp)	Peroxidase
Nrf2 Ab31163, Abcam, Cambridge, UK	Rabbit (polyclonal) IgG, 1:500 dilution	Overnight, 4 °C	Universal antibody by Novolink ^TM^ Leica Biosystems Newcastle ltd, Newcastle Upon Tyne, UK cat. RE7150-K (1 h room temp)	Peroxidase
ElastinAb23747, Abcam, Cambridge, UK	Rabbit (polyclonal), IgG, 1:600 dilution	Overnight, 4 °C	Universal antibody by Novolink ^TM^ Leica Biosystems Newcastle ltd, Newcastle Upon Tyne, UK cat. RE7150-K (1 h room temp)	Peroxidase

**Table 4:** mRNA-Sequencing Software.

Analysis	Software	Version	Parameters	Remarks
Mapping to reference genome	STAR	V2.5	-outFilterMismatchNmax 2	
Quantification	HTSeq	v0.6.1	-m union	
Differential Expression Analysis	DEGseq	v1.34.1	|log_2_Fold change| > 1 && Padj < 0.005	Without biological replicates
	DESeq2	v1.20.0	Padj < 0.05	With biological replicates
GO Enrichment	GOSeq, topGO, hmmscan	v1.34.1	Padj < 0.05	padj < 0.05 were considered significantly enriched
KEGG Pathway Enrichment	KOBAS	v3.0	Padj < 0.05	padj < 0.05 were considered significantly enriched
Reactome Enrichment	clusterProfiler	v3.8.1	Padj < 0.05	padj < 0.05 were considered significantly enriched
Alternative Splicing	rMATS	v4.0.2	-cstat 0.0001 -libType	
Mutation	GATK	v3.8	FS > 30.0 and QD < 2.0	

**Table 5:** Details of primers and probes used for QRT-PCR.

Gene Name	Primers (bp)	Accession Number	Probe Number (Roche)
	FP: Forward Primer		
	RP: Reverse Primer		
Vascular endothelial growth factor A (VEGF-A)	FP: TGCCCGCTGCTGTCTAAT (18)	NM_001025366.2	1
	RP: TCTCCGCTCTGAGCAAGG (18)		
Cluster of differentiation 31 (CD31) (PECAM-1)	FP: CAAAGACAACCCCACTGAAGAC (22)	NM_000442.4	24
	RP: CGCAATGATCAAGAGAGCAATG (22)		
Mast cell tryptase (TPSAB1)	FP: GCGATGTGGACAATGATGAG (20)	NM_003294.3	6
	RP: TCCATTATGGGGACCTTCAC (20)		
Mast cell chymase (CMA1)	FP: ACGGAACTTTGTGCTGACG (19)	NM_001836.4	4
	RP: GGCTCCAAGGGTGACTGTTA (20)		
Elastin (ELN)	FP: CAGCTAAATACGGTGCTGCTG (21)	NM_000501.3	27
	RP: AATCCGAAGCCAGGTCTTG (19)		
Ribosomal protein L32 (RPL32)	FP: CCGTCCCTTCTCTCTTCCTC (20)	NM_001007073.1	10
	RP: TGTCGCAGAGTGTCTTCCAA (20)		
Succinate Dehydrogenase Complex Flavoprotein Subunit A (SDHA)	FP: CAGACCATCTACGGAGCAGAG (21)	NM_004168.3	12
	RP: GATGGGCTTGGAGTAATCGT (20)		

**Table 6:** Table of differentially expressed downregulated genes for Group 1 at week 4. The table gives an example of the first 20 most differentially expressed genes which were downregulated with EGCG compared to placebo in Group 1 at week 4. The yellow highlighted cells represent the hemoglobin genes that were downregulated with EGCG.

Number	Gene ID	Group 1 (EGC—Week 4) Value	Group 1 (Placebo—Week 4) Value	Log2 Fold Change	*p* Value	Padjust	Significance	Gene Name
1	ENSG00000244734	18.56061	225.2407	−3.60115	2.81074 × 10^−53^	5.78282 × 10^−49^	TRUE	HBB
2	ENSG00000188536	4.112765	40.62404	−3.30415	3.67202 × 10^−28^	3.77741 × 10^−24^	TRUE	HBA2
3	ENSG00000022556	0.393985	3.272487	−3.05417	1.70303 × 10^−14^	1.16794 × 10^−10^	TRUE	NLRP2
4	ENSG00000125740	2.008868	5.408491	−1.42884	1.91979 × 10^−9^	5.64253 × 10^−6^	TRUE	FOSB
5	ENSG00000269821	0.067574	0.230412	−1.76967	1.21023 × 10^−7^	0.000177853	TRUE	KCNQ1OT1
6	ENSG00000188487	1.146598	3.394829	−1.56598	3.31126 × 10^−7^	0.000454173	TRUE	INSC
7	ENSG00000235790	2.764742	10.97189	−1.98859	3.77457 × 10^−7^	0.000485363	TRUE	RP11-73M7.6
8	ENSG00000206172	0.363042	3.016038	−3.05445	6.16103 × 10^−7^	0.000667142	TRUE	HBA1
9	ENSG00000153404	1.660928	4.729576	−1.50972	9.92432 × 10^−7^	0.001020915	TRUE	PLEKHG4B
10	ENSG00000251179	0.946775	4.310849	−2.18688	3.62523 × 10^−6^	0.002983423	TRUE	TMEM92-AS1
11	ENSG00000143127	7.800331	19.39026	−1.31372	4.01682 × 10^−6^	0.003178539	TRUE	ITGA10
12	ENSG00000123500	9.860847	24.65212	−1.32193	1.07717 × 10^−5^	0.007641981	TRUE	COL10A1
13	ENSG00000214548	87.26785	236.9831	−1.44126	1.36264 × 10^−5^	0.009043557	TRUE	MEG3
14	ENSG00000280434	0.051907	0.219281	−2.07878	1.66595 × 10^−5^	0.010386463	TRUE	RP4-671O14.6
15	ENSG00000009694	1.286276	2.949952	−1.19749	1.94901 × 10^−5^	0.011234653	TRUE	TENM1
16	ENSG00000121904	2.304434	5.345737	−1.21398	1.86173 × 10^−5^	0.011234653	TRUE	CSMD2
17	ENSG00000153707	7.23834	13.25146	−0.87242	1.96582 × 10^−5^	0.011234653	TRUE	PTPRD
18	ENSG00000198796	4.718508	9.722793	−1.04304	2.37611 × 10^−5^	0.012864772	TRUE	ALPK2
19	ENSG00000157680	2.948852	7.57578	−1.36124	2.89488 × 10^−5^	0.014679513	TRUE	DGKI
20	ENSG00000271811	0.310289	1.285483	−2.05062	3.09708 × 10^−5^	0.015171256	TRUE	RP1-79C4.4

**Table 7:** Uninjured skin analysis. Table displaying the percentage differences between EGCG treated uninjured skin and placebo treated uninjured skin in Groups 1 and 2 pre-emptive priming groups for different parameters measured. Group 1 (Pre-injury: pre-emptive priming 7 days), Group 2 (Pre-injury: pre-emptive priming 3 days).

Uninjured Skin Analysis	Parameter	Group 1 (%)	Group 2 (%)
Non-invasive device analysis	FLPI	−5	−8
	D-OCT	−22	−24
	Elastin	0.4	0.1
	HFUS	0.6	0.4
Immunohistochemical analysis	MCT	−15	−7
	MCC	−16	−14
	CKIT	−17	−15
	FcεRI	−17	−3
	Langerin	−4	−5
	CD31	−10	−10
	VEGF-A	−11	−8
	HO−1	4	8
	NRF2	6	−2
	Elastin	7	3

## Data Availability

All data generated or analyzed during this study are included in this published article. RNA sequencing data has been deposited to Gene Expression Omnibus (GEO accession GSE152781).

## Supplementary Materials

The following are available online at https://www.mdpi.com/article/10.3390/pharmaceutics13040510/s1, Table S1: CONSORT 2010 checklist of information to include when reporting a randomized trial.

## Abbreviations

CD31	Cluster of differentiation 31
CO2	Carbon dioxide
D-OCT	Dynamic Optical Coherence tomography
EGCG	Epigallocatechin-3-gallate
FcεRI	Fc epsilon RI
FDR	False Discovery Rate
FLPI	Full-field laser perfusion imaging
GO	Gene Ontology
HBA	Hemoglobin subunit alpha
HBB	Hemoglobin subunit beta
HFUS	High frequency ultrasound
HIF-1α	Hypoxia-inducible factor 1-alpha
HO-1	Hemeoxygenase-1
IL-8	Interleukin 8
KEGG	Kyoto Encyclopedia of Genes and Genomes
MAPK	Mitogen-activated protein kinase
MCC	Mast cell chymase
MCT	Mast cell tryptase
MMP	Matrix metalloproteinase
mTOR	Mammalian target of rapamycin
NRF2	Nuclear factor erythroid 2-related factor 2
PDGF	Platelet-derived growth factor
PI3K	Phosphoinositide 3-kinase
QRT-PCR	Quantitative reverse transcriptase polymerase chain reaction
RNA	Ribonucleic acid
ROS	Reactive oxygen species
RPKM	Reads Per Kilobase of exon model per Million mapped reads
STAT3	Signal transducer and activator of transcription 3
VEGF-A	Vascular endothelial growth factor A
